# Blocking LAIR1 signaling in immune cells inhibits tumor development

**DOI:** 10.3389/fimmu.2022.996026

**Published:** 2022-09-21

**Authors:** Jingjing Xie, Xun Gui, Mi Deng, Heyu Chen, Yuanzhi Chen, Xiaoye Liu, Zhiqiang Ku, Lingxiao Tan, Ryan Huang, Yubo He, Bruce Zhang, Cheryl Lewis, Kenian Chen, Lin Xu, Jian Xu, Tao Huang, X. Charlene Liao, Ningyan Zhang, Zhiqiang An, Cheng Cheng Zhang

**Affiliations:** ^1^ Department of Physiology, University of Texas Southwestern Medical Center, Dallas, TX, United States; ^2^ Texas Therapeutics Institute, Brown Foundation Institute of Molecular Medicine, University of Texas Health Science Center, Houston, TX, United States; ^3^ Harold C. Simmons Comprehensive Cancer Center, University of Texas Southwestern Medical Center, Dallas, TX, United States; ^4^ Department of Population and Data Sciences, University of Texas Southwestern Medical Center, Dallas, TX, United States; ^5^ Department of Pediatrics, University of Texas Southwestern Medical Center, Dallas, TX, United States; ^6^ Children’s Medical Center Research Institute, University of Texas Southwestern Medical Center, Dallas, TX, United States; ^7^ Immune-Onc Therapeutics, Inc, Palo Alto, CA, United States

**Keywords:** antibody, cancer immunotherapy, RNAseq, T cell, myeloid cells, natural killer cell, humanized mice, LAIR1

## Abstract

The current immune checkpoint blockade therapy has been successful in treating some cancers but not others. New molecular targets and therapeutic approaches of cancer immunology need to be identified. Leukocyte associated immunoglobulin like receptor 1 (LAIR1) is an immune inhibitory receptor expressing on most immune cell types. However, it remains a question whether we can specifically and actively block LAIR1 signaling to activate immune responses for cancer treatment. Here we report the development of specific antagonistic anti-LAIR1 monoclonal antibodies and studied the effects of LAIR1 blockade on the anti-tumor immune functions. The anti-LAIR1 antagonistic antibody stimulated the activities of T cells, natural killer cells, macrophages, and dendritic cells *in vitro*. The single-cell RNA sequencing analysis of intratumoral immune cells in syngeneic human LAIR1 transgenic mice treated with control or anti-LAIR1 antagonist antibodies indicates that LAIR1 signaling blockade increased the numbers of CD4 memory T cells and inflammatory macrophages, but decreased those of pro-tumor macrophages, regulatory T cells, and plasmacytoid dendritic cells. Importantly, the LAIR1 blockade by the antagonistic antibody inhibited the activity of immunosuppressive myeloid cells and reactivated T cells from cancer patients *in vitro* and impeded tumor metastasis in a humanized mouse model. Blocking LAIR1 signaling in immune cells represents a promising strategy for development of anti-cancer immunotherapy.

## Introduction

The breakthrough of the immune checkpoint blockade therapy has revolutionized cancer treatment. However, the existing immune checkpoint blockade approach is only effective in treating some cancers (1). To more effectively treat cancer patients, new molecular targets and therapeutic approaches must be identified.

ITIM-containing receptors are emerging immune checkpoint targets in cancer treatment (2). Leukocyte-associated immunoglobulin-like receptor 1 (LAIR1, or CD305) is a type I transmembrane glycoprotein that contains one extracellular Ig-like domain and two intracellular ITIMs. LAIR1 is expressed on most hematopoietic lineages, including monocytes, macrophages, dendritic cells (DCs), natural keller (NK) cells, and many T and B cell populations (3–5). Its extracellular domain binds to Gly-Pro-Hyp collagen repeats, and its ITIMs recruit phosphatases SHP-1 and SHP-2. Collagens, C1q, MBL, surface protein-D (SP-D), Rifins, and Colec12, have been reported as ligands for LAIR1 (6–11).

As an immune inhibitory receptor, LAIR1 appears to be dispensable for normal hematopoiesis (12–14). Nevertheless, LAIR1 was reported to have the function on regulating immune system balance and protecting tissue damages against a hyperactive immune response or autoimmune dysfunction (4). The roles of LAIR1 in different types of cancer have been studied. Two independent studies by us and Müschen’s group using *in vitro* and xenograft experiments showed that LAIR1 deficiency retards development of acute myeloid leukemia (AML) and Philadelphia Chromosome positive acute lymphoblastic leukemia (Ph^+^ B-ALL) (13, 14). The LAIR1/SHP-1/CAMKI/CREB axis supports AML stem cells (14). In Ph^+^ B-ALL cells, LAIR1 mediates dephosphorylation of Syk by SHP-1 and SHIP, which enables the negative selection of overactivated B cells during B-ALL cell transformation (13). In chronic lymphoblastic leukemia (CLL), down-regulation of LAIR1 correlates with increased risk of disease (15, 16). Antibody engagement with LAIR1 blocks AKT and NF-κB activation in CLL cells, leading to decreased cell proliferation (16). Although LAIR1 was reported to be abnormally upregulated on certain solid cancer cells (17–21), its main function during solid cancer development may result from its inhibition of activation of multiple types of immune cells (9, 22–25). However, it was unclear whether we can specifically and actively antagonize LAIR1 signaling to achieve anti-cancer functions.

In this study, we report the generation of anti-LAIR1 humanized antibodies, and focused on studying the function of h219, an antagonist antibody, in anti-tumor immune responses. We demonstrated that h219 stimulates the activities of multiple immune cell types using *in vitro* cultures and single-cell RNA sequencing analysis of intratumoral immune cells in human LAIR1 transgenic mice. Importantly, treatment with h219 significantly inhibited the activity of immunosuppressive myeloid cells and restimulated T cell proliferation from cancer patients *in vitro* and impeded tumor metastasis in humanized mice. These results suggest that blocking LAIR1 signaling with antagonist antibodies represents a new attractive anti-cancer strategy.

## Materials and methods

### Patients

We collected blood samples from solid cancer patients through UT Southwestern Tissue Management Shared Resource. Informed consent was obtained under protocols reviewed and approved by the Institutional Review Board at UTSW. The samples were distributed to the laboratory in a de-identified fashion, and the proposed study is not considered human research.

### Mice

C57 BL/6J and NOD-SCID IL2Rγ-null-SGM3 (NSG-SGM3) mice (Jax #013062 were purchased from Jackson Laboratory and maintained at the animal core facility of University of Texas Southwestern Medical Center (UTSW). Hematopoietic-specific human LAIR1 transgenic mice were generated at the Transgenic Core Facility at UT Southwestern. The LAIR1 knockout mice (12) were a kind gift from Dr. John E. Coligan from NIH. Animal work described in this manuscript has been approved and conducted under the oversight of the UT Southwestern Institutional Animal Care and Use Committee (IACUC). The same sex- and age-matched (4–8 weeks) mice were used and randomly allocated to each group; and for tumor size measurement, experimenters were blinded to the treatment conditions of the mice. For the subcutaneous tumor model, the tumor size was calculated as (length × width × width)/2.

### Cell lines

HEK293F and CHO cell lines were obtained from Life Technologies (Carlsbad). Human monocytic AML cell lines (THP-1, MV4-11 and U937) were obtained from ATCC and maintained in a humidified atmosphere of 5% CO_2_ at 37°C, in media suggested by ATCC supplemented with fetal bovine serum (FBS) (HyClone) and 100 U/mL penicillin and 100 μg/mL streptomycin (Life Technologies). All cell lines were routinely tested using a mycoplasma-contamination kit (R&D Systems).

### Flow cytometry

Cells were washed in FACS medium (PBS containing 2% FBS), and stained with primary antibodies including anti-human CD14-APC (eBioscience, 61D3, 1:100), anti-human CD4-APC (eBioscience, RPA-T4, 1:100), anti-human CD3-FITC (BioLegend, HIT3a, 1:100), anti-human CD8-PE (BD Pharmingen, 555367, 1:100), anti-human CD69-APC (BioLegend, FN50, 1:100), anti-human CD86-PE (BioLegend, IT2.2, 1:100), anti-CD206-APC (eBioscience, 19.2, 1:100), anti-CD11c (Biolegend, Bu15, 1:100), anti-hLAIR1 (eBioscience, NKTA255, 1:100), anti-CD45 (Biolegend, 2D1, 1:100), anti-CD25 (Biolegend, BC96, 1:100), or anti-CD127 (Biolegend, A019D5, 1:100). Flow data were analyzed by Flowjo software. Propidium iodide (PI) staining was used to exclude dead cells in analysis.

### Chimeric receptor reporter assay

The LAIR1 chimeric receptor reporter cells were developed by us as previously described (14). A competition assay was used to screen LAIR1 antagonist antibodies. Briefly, collagen (10 μg/ml) was precoated on 96-well plates at 37°C for 3 hours. After washes with PBS twice, 4 x 10^4^ LAIR1 reporter cells were seeded into each well. Meanwhile, indicated LAIR1 antibodies were added into culture media. After culture for 20 hours, the percentage of GFP^+^ reporter cells was measured by flow cytometry.

### Tumor experiment in Vav-Cre LAIR1-transgenic mice

5x10^5^ B16 tumor cells were injected subcutaneously into each mouse (VAVcre+/-hLAIR1+/- mLAIR1-/- in C57BL/6 background) at day 0. Five days later, mice were randomized into two groups based on tumor size, followed by iv injection of control or anti-hLAIR1 blocking antibodies (10 mg/kg) every four days. After 3 doses of antibody administration, mice were euthanized at day 16.

### Tumor experiment in humanized tumor mice

5x10^4^ human cord blood CD34^+^ cells (STEMCELL #70008) were transplanted into 250cGy sublethally irradiated NSG-SGM3 mice (Jax #013062); after 11 weeks we detected significant human CD45^+^ chimerism (with human myeloid and T cells) in blood of mice as described (26–29). We then *subcutaneously* implanted 1x10^6^ MDA-MB-231 human breast cancer cells with 50% Matrigel (Corning #354234) at week 14. After tumor size reached 50-60 mm^3^ between week 15-16, we randomized the mice into indicated groups and started to treat the mice with control or anti-LAIR1 blocking mAbs. Liver metastasis was observed at another 9 weeks post-tumor implantation when we euthanized the mice.

### Human normal monocytes, macrophages, and dendritic cells

Human normal monocytes (CD14^+^ cells) were isolated by the AutoMACS Pro Separation System (Miltenyi Biotech) from the mononuclear cell fraction of normal peripheral blood. In brief, buffy coat was purchased from Interstate Blood Bank and the mononuclear cell layer was separated by Ficoll Hypaque (17144003, GE Lifesciences) density gradient. Mononuclear cells were treated with red blood cell lysis buffer to remove red blood cells and then incubated with anti-CD14 microbeads (130-050-201, Miltenyi Biotech) for 15 min at 4 °C. CD14-positive cells were then isolated using the positive selection program according to the manufacturer’s protocol.

The protocol of M1 and M2 differentiation was modified from those in (30, 31). To develop the M1 macrophages, 1x10^5^ CD14^+^ cells were cultured in RPMI 1640 medium (R8758-500ml, Sigma) supplemented with 10% fetal bovine serum (F0926, Sigma), 2 mM L-alanine-L-glutamine (SH3003402, Fisher), and 25 ng/ml GM-CSF (300-03, PeproTech) per well of a 96-well plate for 6 days. Fresh medium was replaced every 3 days. Afterwards, cells were continually cultured in RPMI 1640 medium (R8758-500ml, Sigma) supplemented with 10% fetal bovine serum (F0926, Sigma), 2 mM L-alanine-L-glutamine (SH3003402, Fisher), 100 ng/ml LPS (L4516, Sigma), and 50 ng/ml Interferon-gamma (300-03, PeproTech) for another 2 days.

To develop the M2a macrophages, 1x10^5^ CD14^+^ cells were cultured in macrophage-SFM (12065-074, Gibco) supplemented with 2 mM L-alanine-L-glutamine (SH3003402, Fisher), 100ng/ml M-CSF (11792-H08H-20, Sino Biological) per well of a 96-well plate for 6 days. Fresh medium was replaced every 3 days, and 20 ng/ml human IL-4 (11846-HNAE-1, Sino Biological) was added on day 3. Polarization started on day 6 with the medium used on day 3 supplemented with 100 ng/ml LPS (L4516, Sigma) and lasted for another 24 hours.

The protocol of dendritic cell differentiation was modified from those in (32, 33). 1x10^5^ CD14^+^ cells were cultured in RPMI 1640 medium (R8758-500ml, Sigma) supplemented with 10% fetal bovine serum (F0926, Sigma), 2 mM L-alanine-L-glutamine (SH3003402, Fisher), 50 ng/ml GM-CSF (300-03, PeproTech) and 25 ng/ml human IL-4 (11846-HNAE-1, Sino Biological) per well of a 96-well plate for 6 days; fresh medium was replaced every 3 days. Cells were stimulated on day 7 with RPMI 1640 medium (R8758-500ml, Sigma) supplemented with 10% fetal bovine serum (F0926, Sigma), 2 mM L-alanine-L-glutamine (SH3003402, Fisher), 100 ng/ml LPS (L4516, Sigma), and 1000 U/ml Interferon-gamma (300-03, PeproTech) for 48 hours. Then mature dendritic cells were harvested and counted.

### Mixed lymphocyte reaction

Mixed lymphocyte reaction was carried out in the 96-well plate essentially as described (34). Human allogeneic pan T cells (PB009-1F, Allcells) were stained with Carboxy fluorescein succinimidyl ester (CFSE) (C34554, Thermo Fisher Scientific), and mixed with 3 x10^5^ mature DCs at the ration of 1:1 in a total volume of 200 μl of RPMI 1640 supplemented with 10% FBS. CFSE-based proliferation of T cells was measured by flow cytometry.

### T cells

Anti-CD3 antibody (Biolegend, OKT3) was used to stimulate T cell proliferation essentially as described (26, 27, 35). 1 µg/ml anti-CD3 antibody diluted in PBS was pre-coated on the bottom of a 96-well plate. 1x10^5^ fresh isolated PBMCs or pan T cells were plated in RPMI 1640 medium (R8758-500ml, Sigma) supplemented with 10% fetal bovine serum (F0926, Sigma) and 100 U/ml IL-2 (200-02, PeproTech, for pan T cells only) per well of the pre-coated 96-well plate for 3-5 days. Activation and proliferation of T cells were measured by flow cytometry.

### MDSC/T coculture

The CD11b^+^ HLA-DR^dim^ cells from indicated cancer patients were cocultured with CFSE-stained autologous T cells (E:T =1) for 5 days, treated with 10 µg/mL LALA-PG mutated control antibodies or h219 anti-LAIR1 blocking antibody. 12.5 μL/mL of ImmunoCult™ Human CD3/CD28 T Cell Activator (STEMCELL Technologies, Cat# 10971) was used to induce the activation of CD3^+^ T cells.

### Statistical analyses

Two-tailed Student t-test and Mann-Whitney log-rank test were used for comparison of two groups. One-way ANOVA test was employed for comparison of three groups. *In vitro* data were presented as mean ± SEM. In all figures, * indicates p < 0.05; ** indicates p < 0.01, *** indicates p < 0.001, **** indicates p < 0.0001.

## Results

### LAIR1 expression in immune cells in tumor microenvironment

It is known that LAIR1 is expressed on most hematopoietic lineages of cells. To understand whether LAIR1 is more highly expressed by certain immune cells in the human tumor microenvironment (TME), we searched the Cancer Genome Atlas (TCGA) database and analyzed the correlation between the expression of LAIR1 and those of several immune cell markers. We found that LAIR1 expression was generally highly correlated with CD33 (a marker for myeloid cells) and CD4 (a marker for T cells) in most cancers except liver cancer (**Supplementary Figure 1** and **Supplementary Table 1**). This result suggests that LAIR1 is highly expressed by certain myeloid cells and T cells in TME of many cancers, and it may therefore be particularly important to study the function of LAIR1 in these immune cells in TME.

### Generation of an anti-LAIR1 blocking mAb

To test the hypothesis that LAIR1 blockade in immune cells induces anti-cancer immune responses, we generated a panel of anti-LAIR1 monoclonal antibodies from rabbits (**Supplementary Figure 2A**). We measured the EC_50_ for these antibodies using ELISA which were at the low nanomolar range (0.05-0.3 nM) (**Supplementary Figure 2B**).

To examine whether these anti-LAIR1 rabbit mAbs block LAIR1 activation by collagen I, a functional ligand of LAIR1, we screened the mAbs using the LAIR1 chimeric receptor reporter cells we previously established (14). Twenty-four antibodies were found to be able to neutralize the collagen I-mediated LAIR1 activation (**Supplementary Figure 2C**). GPVI, a protein containing 2 extracellular Ig-like domains, has different functions than LAIR1 yet possesses a similar collagen-binding property (36). We further identified 19 LAIR1 blockers that are specific to LAIR1 and do not cross react with GPVI (**Supplementary Figure 2D** **)**, and selected 7 (**Supplementary Figure 2E** **)** for further experiments. All 7 mAbs showed strong ligand blocking activities (**Supplementary Figure 3A**), with Kd values for binding to LAIR1 were from 0.44 nM to 1.31 nM as determined by an Octet RED96 binding assay (**Supplementary Figure 3B**). To group these 7 mAbs by their binding epitopes, we performed a sandwich epitope binning assay with an Octet RED96. Two bins, Bin1 and Bin2, were identified for these 7 LAIR1 blockers (**Supplementary Figure 4**). We selected R-219 as the lead mAb based on its overall high LAIR1 binding activity (**Supplementary Figures 2F, G**, **3**) and potent blocking efficacy against collagen-induced LAIR1 activation (**Supplementary Figure 3A**).

**Figure 1:**
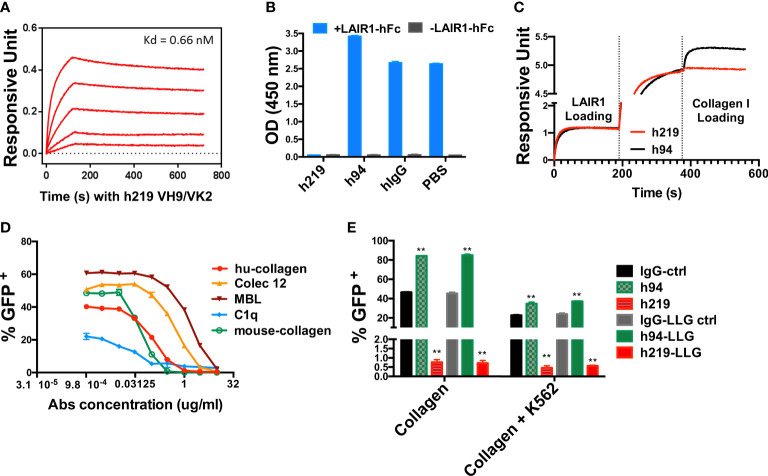
Humanized 219 (h219) is an antagonist anti-LAIR1 monoclonal antibody. **(A)** Affinity of h219 (VH9/VK2) to human LAIR1 was determined by Octet RED96. ForteBio’s data analysis software was used to fit the data to a 1:1 binding model to extract an association rate and dissociation rate. The Kd was calculated using the ratio k_dis_/k_on_. **(B)** h219 blocks LAIR1 and collagen I interaction in ELISA. Collagen I was coated on 96-well plate at 1 μg/ml. Pre-incubated LAIR1-hFc (2 μg/ml) and h219, h94, or hIgG (5 μg/ml) were added to the plate after blocking with 5% non-fat milk. HRP conjugated goat anti-human IgG Fc was used to detect the binding of LAIR1-hFc to Collagen I. **(C)** h219 blocks LAIR1 and Collagen I interaction performed by Octet RED96. NTA biosensors were used to capture his tagged human LAIR1 recombinant protein (20 μg/ml). After loading of h219 or control antibody h94 (40 μg/ml), the collagen I (80 μg/ml) binding signal was detected. **(D)** h219 blocks LAIR1 activation induced by the indicated molecules as determined by the LAIR1 chimeric receptor reporter cell assay. **(E)** Comparison of the activities of control, h94, and h219 antibodies with wildtype Fc or with LALA-PG mutation in Fc that does not have Fc-mediated effector function (see **Supplementary Figure 7**) to block collagen-induced LAIR1 activation. Immobilized collagen (left) or collagen stably expressed on K562 cells (right) was used to induce LAIR1 activation in LAIR1 chimeric receptor reporter cells. One-way ANOVA test for IgG-ctrl, h94, and h219, p<0.0001. One-way ANOVA test for IgG-LLG ctrl, h94-LLG, and h219-LLG, p<0.0001, ** p < 0.01.

For potential therapeutic development, we used a CDR grafting strategy to humanize R-219, converting it into a human IgG1 subclass with a kappa light chain (**Supplementary Figures 5A, B**). Afterwards, we further engineered the heavy chain CDR3 and light chain CDR3 to remove potential deamination residues (**Supplementary Figures 5C-E**). We selected the heavy and light chain combination VH9/VK2 as our final version of humanized R219 (h219). Next, we measured the binding affinity of h219 to LAIR1 by Octet RED96 and the ligand blocking activity using LAIR1 chimeric receptor reporter assay. The Kd of h219 binding to LAIR1 as measured by Octet RED96 was 0.66 nM (**Figure 1A**), comparable to the Kd of the parental mAb R-219 (0.70 nM). We also tested the collagen blocking activity of h219 in competition ELISA and Octet system. As shown in **Figures 1B, C**, h219 totally blocked the interaction of LAIR1 with collagen I. In contrast, h94, another humanized anti-LAIR1 antibody, did not block but rather enhanced the collagen I-LAIR1 interaction, suggesting that h94 strengthens the agonist activity of collagen I.

Next, we employed the LAIR1 chimeric receptor reporter cells to determine the ligand-blocking capacities of h219 and found that h219 was able to antagonize LAIR1 activation induced by several reported ligands including human collagen I, mouse collagen I/III, C1q, Colec12, and MBL (**Figure 1D**). Together, we applied a number of criteria to screen the antagonist antibodies against LAIR1: a) The antibody has a high specific binding affinity to human LAIR1 as determined by ELISA, Octet RED96, and flow cytometry-based assays (data not shown). b) We also ensured the specificity of the antibody so that it does not bind GPVI, LILRB1-5, LILRA1-6, or mouse LAIR1 (data not shown). c) The antibody blocks LAIR1 activation induced by various ligands. In addition, the antibody blocks collagen-induced LAIR1 activation in the presence of K562 cells (**Figure 1E**) which express FcR that may crosslink Fc-containing anti-LAIR1, a situation that may happen *in vivo* when different types of cells coexist in a microenvironment. Our initial results suggest that h219 is an attractive antagonist antibody against human LAIR1.

We further evaluated the direct effect of h219 on LAIR1^+^ cells and found that h219 did not inhibit proliferation (**Supplementary Figures 6A, B** **)**, induce apoptosis (**Supplementary Figures 6C, D** **)**, or block migration (**Supplementary Figures 6E, F** **)** of these cells *in vitro*. h219 does induce antibody-dependent cellular cytotoxicity (ADCC) in LAIR1^+^ cells (**Supplementary Figure 7A**). To exclude the Fc-mediated effector functions, we generated LALA-PG mutation (37) for h219 (as h219-LLG) that does not induce Fc-mediated effector functions including ADCC but has the comparable binding ability to LAIR1 as the wild-type h219 (**Supplementary Figures 7B, C** **)**. h219-LLG was used in the subsequent experiments.

### Effects of LAIR1 inhibition on T cell activation and proliferation

**Figure 2:**
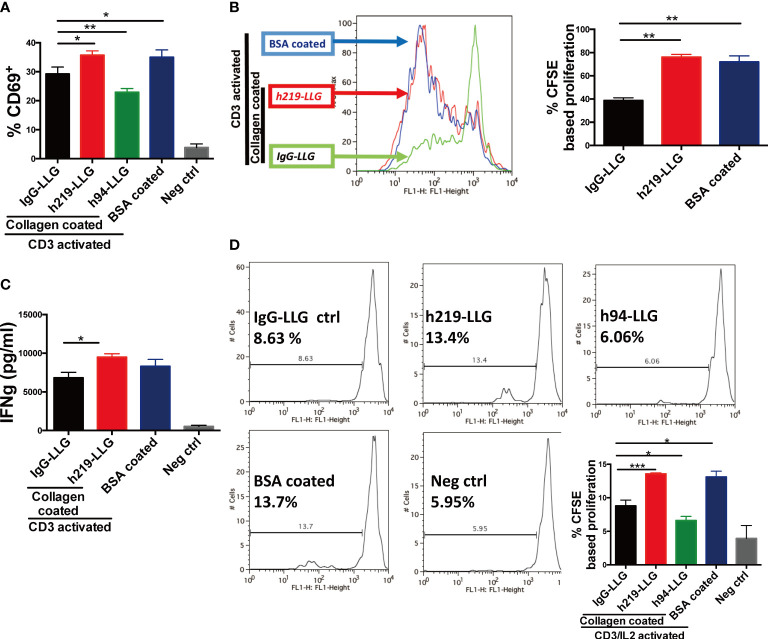
Inhibition of LAIR1 enhances primary T cell activation and proliferation. **(A-C)** Anti-LAIR1 h219 promoted T cell activation in human PBMCs stimulated with 1 μg/ml anti-CD3 antibody (OKT3). Cell surface CD69 was detected at 12 hours **(A)**. CFSE based CD3^+^ cell proliferation was detected on day 3 **(B)**. IFN-γ secretion was detected by ELISA on day 5 **(C)**. **(D)** h219 promoted activation of purified T cells. Pan T cells were activated with 1 μg/ml anti-CD3 antibody (OKT3) and IL2. CFSE based T cell proliferation was detected on day 3. In **(A)** one-way ANOVA test for IgG-ctrl, h94, and h219, p<0.0006. In **(D)** one-way ANOVA test for IgG-ctrl, h94, and h219, p<0.0001. As Statistical Analysis in Methods stated: * indicates p < 0.05; ** indicates p < 0.01, *** indicates p < 0.001.

We sought to determine whether blocking LAIR1 signaling can stimulate the activities of immune cells. Because our analysis suggests that LAIR1 is highly expressed in T cells in TME (**Supplementary Table 1** and **Supplementary Figure 1**), we started to assess the effect of LAIR1 signaling blockade on T cells. To this end, we stimulated human PBMCs with coated anti-CD3 antibody (OKT3) (38). The activation of LAIR1 by immobilized collagen significantly decreased the expression of CD69, an early marker for T cell activation (39), on T cells compared to the immobilized BSA control (**Figure 2A**). This reduction of CD69 was restored by the treatment of the antagonist anti-LAIR1 antibody h219-LLG. By contrast, h94, an anti-LAIR1 agonist antibody (**Figures 1B, C** **)**, further downregulated CD69 on T cells (**Figure 2A**). This result is consistent with those from the T cell proliferation assay and cytokine analysis (**Figures 2B, C** and **Supplementary Figure 8**). To exclude the possible influence of other cells in PBMCs on T cell activation and proliferation, we further tested the effect of h219 and h94 on purified human T cells stimulated with OKT3 and 100 U/ml IL-2 (40). Again, in the presence of immobilized collagen, the treatment of h219-LLG antagonist antibody induced whereas h94 agonist antibody reduced T cell proliferation (**Figure 2D**). Our results indicate that LAIR1 blockade by the antagonist antibody reactivates T cells.

### Effects of LAIR1 inhibition on macrophage differentiation

**Figure 3:**
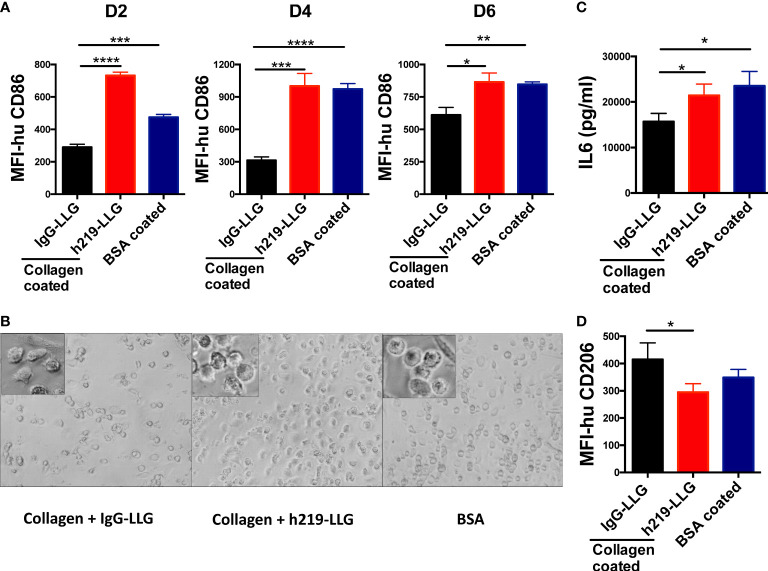
Inhibition of LAIR1 promotes differentiation and cytokine secretion of *in vitro* differentiated human macrophages. **(A, B)** M1 macrophages were *in vitro* differentiated from CD14^+^ monocytes isolated from fresh human PBMCs. Cells were treated with collagen-1 and antibodies from day 0 when differentiation started. MFI of CD86 was detected on day 2, 4, and 6 **(A)**. Morphology of cells on day 4 **(B)**. **(C)** M0 macrophages were polarized with IFN-γ and LPS, meanwhile treated with collagen-1 and antibodies. IL6 in supernatant was determined at 48 hours after polarization. **(D)** M2a macrophages were *in vitro* differentiated from CD14^+^ monocytes isolated from fresh human PBMCs. Collagen-1 and antibodies were included in the differentiation from day 0. MFI of CD206 was detected on day 8, when was 1 day after polarization. As the Statistical Analysis section of Methods describes, * indicates p < 0.05; ** indicates p *< 0.01, *** indicates p < 0.001, **** indicates p < 0.0001.

Besides T cells, LAIR1 was suggested to be highly expressed in myeloid cells in TME (**Supplementary Table 1** and **Supplementary Figure 1**). It was reported that LAIR1 crosslinking inhibited the polarization (41) and the inflammatory phenotype of macrophages *in vitro* (42). To study whether LAIR1 blockade regulates the differentiation and polarization of macrophages, we treated the freshly isolated CD14^+^ monocytes with GM-CSF and anti-LAIR1-LLG or control antibodies. GM-CSF treatment of human monocytes led to differentiation of M1 macrophages with a “proinflammatory” cytokine profile (43) (**Supplementary Figure 9A**). The surface expression of CD86, an M1 marker, was significantly elevated by h219-LLG in the presence of immobilized collagen (**Figure 3A**). Concordantly, compared to the collagen treated controls, h219 treatment in the presence of immobilized collagen led to the more round and flattened morphology of M1 macrophages (44) (**Figure 3B**). Then, after 6 days of treatment by GM-CSF and anti-LAIR1-LLG or control antibodies, the CD14^+^ cells were further polarized with IFN-γ and LPS for another 2 days. The expression of IL-6, a pro-inflammatory cytokine, was higher in the positive control group (coated BSA) than in the collagen treated group (**Figure 3C**), suggesting LAIR1 activation by collagen inhibits the differentiation to M1 macrophages. This inhibition of M1 differentiation by the LAIR1 ligand collagen was reversed by h219-LLG (**Figure 3C**).

We further investigated whether LAIR1 blockade regulates the differentiation of monocytes to the anti-inflammatory M2a macrophages (**Supplementary Figure 9A**). The treatment of h219-LLG significantly reduced the level of CD206, a M2a marker (**Figure 3D**). Together, our results demonstrate that blocking of LAIR1 signaling stimulates M1 but inhibits M2 macrophage polarization.

### Effects of LAIR1 inhibition on dendritic cell differentiation and function

**Figure 4:**
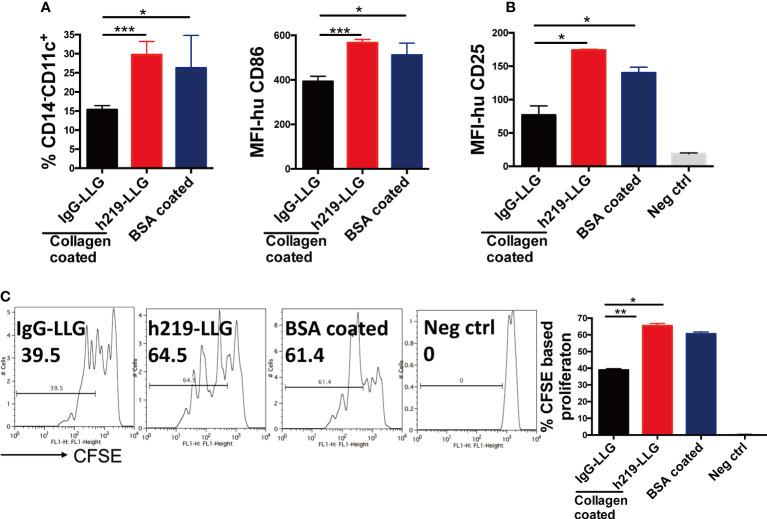
Inhibition of LAIR1 enhances dendritic cell differentiation and dendritic cell- induced T cell proliferation in MLR assay. **(A)** CD14^+^ monocytes isolated from fresh human PBMCs were cultured with GM-CSF and IL-4 for 2 days. The expression of CD11c on CD14 negative population and CD86 were detected. **(B, C)** Matured DCs were mixed with allogenic T cells in the MLR assay. Surface CD25 **(B)** and CFSE based cell proliferation were determined by flow cytometry at 72 hours **(C).** As Statistical Analysis in Methods stated: * indicates p < 0.05; ** indicates p < 0.01, *** indicates p < 0.001.

It was shown that C1q, a ligand of LAIR1, blocked GM-CSF– and IL-4–induced DC differentiation from monocytes (7). We sought to test whether LAIR1 blockade regulates the differentiation and function of DCs. We used collagen to activate LAIR1 during the differentiation from monocytes to DCs and administered h219 to inhibit LAIR1 signaling. The DC differentiation is accompanied with a decrease of the level of the monocytic marker CD14 and increases of the expression of DC markers such as CD11c and CD86 (**Supplementary Figure 9B**). In the presence of collagen, the percentage of CD14^-^ CD11c^+^ cells was down-regulated. This down-regulation was reversed by the h219-LLG treatment (**Figure 4A**). A similar trend happened to CD86^+^ cells (**Figure 4A**). We further conducted a mixed lymphocyte reaction (MLR) experiment to measure the function of DCs. h219-LLG treatment led to significant increase of proliferation of allogeneic T cells and the surface expression of CD25 (a T cell activation marker) in this MLR compared to control conditions (**Figures 4B, C** and **Supplementary Figure 9**).

### Effects of LAIR1 inhibition on NK cell function

Besides myeloid cells and T cells, it is known that NK cells are LAIR1^+^ (3). Indeed, treatment of h219-LLG increased the cytotoxic activities of NK cells against the leukemia cell line K562 **(** **Supplementary Figure 10** **)**. Our result indicates that antagonizing LAIR1 signaling increases the cytotoxic activity of NK cells against cancer cells.

### LAIR1 inhibition restored T cell proliferation in coculture of MDSCs and autologous T cells isolated from solid cancer patients

**Figure 5:**
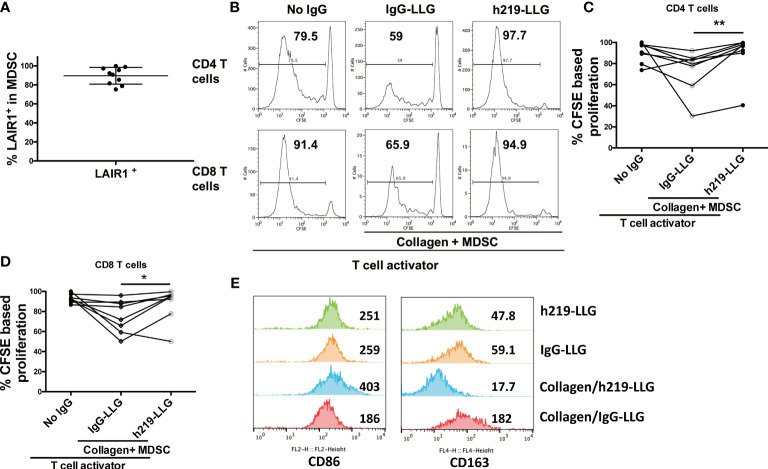
LAIR1 blockade inhibits MDSC activity and reactivates T cells in cancer patients. **(A)** LAIR1^+^% in CD11b^+^ HLA-DR^dim^ cells as enriched MDSCs isolated from prostate cancer patients as determined by flow cytometry. **(B)** Representative histograms show that h219-LLG attenuated the T cell suppressive function of MDSCs from prostate cancer patients in a CSFE assay. The CD11b^+^ HLA-DR^dim^ cells were cocultured with CFSE-stained autologous T cells (E:T =1) for 5 days, treated with 10 µg/mL control or h219-LLG antibodies. **(C, D)** Quantification of the percentages of proliferative CD4^+^ and CD8^+^ T cells treated as indicated. **(E)** Enriched MDSCs from a patient with squamous cell carcinoma were cultured without T cells in the presence or absence of collagen, treated with h219-LLG or control antibodies for 5 days. Shown are MFIs of surface CD86 and CD163 as measured by flow cytometry. As Statistical Analysis in Methods stated: * indicates p < 0.05; ** indicates p < 0.01.

While the above studies focused on LAIR1 function in normal immune cells, we were also interested in asking whether LAIR1 regulates the anti-cancer immunity in cancer patients. To this end, we collected the phenotypic myeloid-derived suppressor cells (MDSCs) and autologous T cells from blood samples from prostate cancer and lung cancer patients (**Supplementary Table 2**). Due to the heterogeneity of MDSCs (45), we enriched CD11b^+^HLA-DR^dim^ cells by autoMACS as phenotypic MDSCs (45, 46). All of the enriched MDSCs expressed LAIR1 as determined by flow cytometry (**Figure 5A**). These cells effectively inhibited the proliferation of autologous T cells in a co-culture system similar to what we have described (26, 27) (**Figures 5B-D**), attesting to their activity as MDSCs. Importantly, h219-LLG reversed the immunosuppressive activity of MDSCs and reactivated T cells (**Figures 5B-D**). To clarify the effect of lAIR1 blockade on MDSC activity, we treated enriched MDSCs isolated from a lung cancer patient with squamous cell carcinoma with h219-LLG or control antibodies in the presence or absence of collagen. h219-LLG treatment increased the expression of activation marker CD86, but decreased that of the immunosuppressive marker CD163 (**Figure 5E**). Our result demonstrates that LAIR1 is functionally expressed on MDSCs and LAIR1 blockade inhibits MDSC activity and reactivates T cells in cancer patients.

### LAIR1 inhibition delayed tumor development *in vivo*

**Figure 6:**
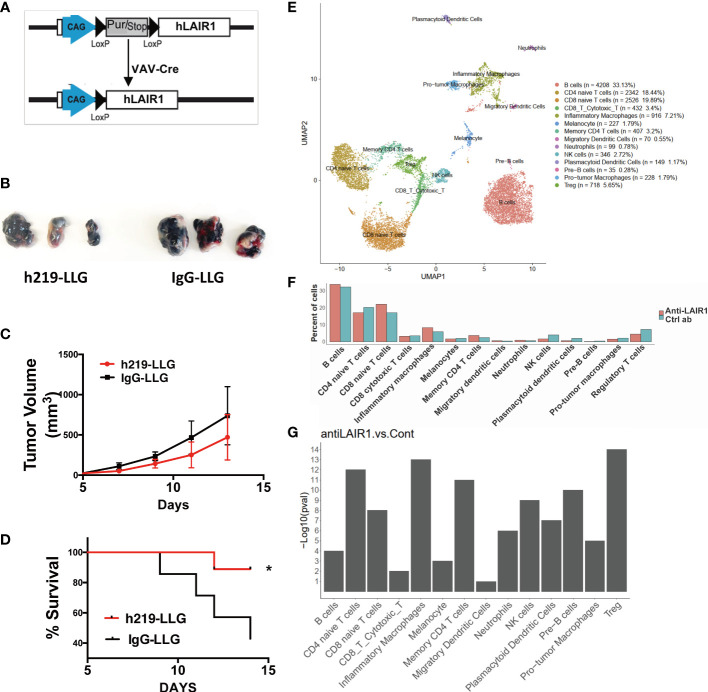
LAIR1 blockade alters immune profiling in tumor microenvironment in a syngeneic LAIR1-transgenic mouse tumor model. **(A)** Schematic of Vav-Cre human LAIR1 transgene in mouse LAIR1 KO background. **(B, C)** Pictures of tumors and tumor volumes in mice treated by h219-LLG and control in a representative experiment. **(D)** Survival curve of mice treated by h219 or control antibodies (n=9). **(E)** Cell type annotation based on unsupervised clustering. **(F)** Comparison of percentages of immune cell types in TME in mice treated by h219-LLG or control antibodies. **(G)** Chi-square test P-values of percentage changes of immune cell types treated by h219-LLG or control antibodies. As Statistical Analysis in Methods stated: * indicates p < 0.05.

We developed hematopoietic-specific (*Vav-cre*) LAIR1-transgenic C57BL/6 mice (**Figure 6A**). To avoid the interference of mouse LAIR1 during tumor development, we bred the Vav-Cre hLAIR1 transgenic mice in the mLAIR1-/- background. The transgenic LAIR1 is expressed on all lineages of hematopoietic cells in these mice (**Supplementary Figure 11**). It is known that mouse collagen can bind and activate human LAIR1 (**Figure 1D**) in this system. h219-LLG antibody prolonged the survival of the transgenic mice implanted with B16 mouse tumor cells (**Figures 6B-D**). To identify what occurred in the immune TME, we performed the single-cell RNA-sequencing (scRNAseq) analysis of mouse CD45^+^ immune cells in tumor tissues (**Figures 6E-G**). With unsupervised clustering, we identified 13 immune cell types (in addition to the contaminating B16 melanocytes), including CD4 naïve T cells, CD4 memory T cells, CD8 naïve T cells, CD8 cytotoxic T cells, regulatory T cells (Tregs), Pre-B cells, B cells, NK cells, inflammatory macrophages, pro-tumor macrophages, neutrophils, migratory dendritic cells, and plasmacytoid dendritic cells (**Figure 6E**). Consistent with our functional results, h219-LLG increased percentages of CD4 memory T cells and inflammatory macrophages, but decreased those of pro-tumor macrophages, Tregs, and plasmacytoid dendritic cells (**Figures 6F, G**).

**Figure 7:**
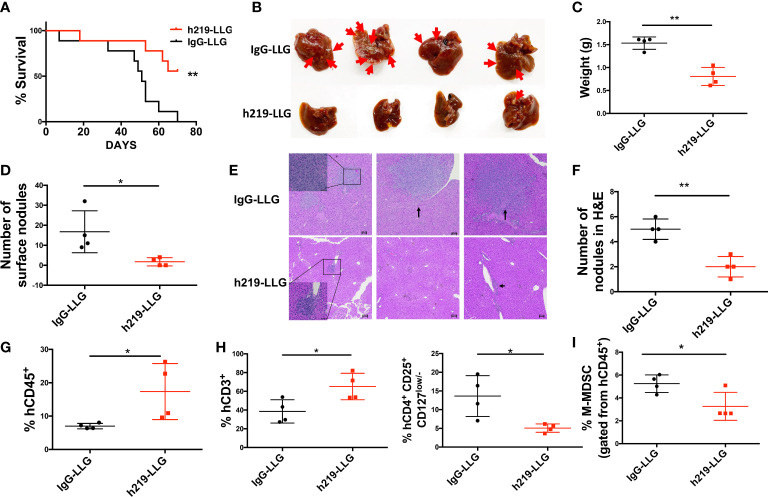
LAIR1 blockade reduces MDA-MB-231 tumor cell metastasis and prolongs survival in human cord blood CD34^+^ cell-reconstituted humanized mice. **(A)** Survival curve of mice treated by h219-LLG or control antibodies (n=9). **(B)** The pictures of livers from mice. Arrows indicate nodules formed by metastasized tumor cells. **(C, D)** The liver weight **(C)** and the number of surface nodules on livers **(D)**. **(E, F)** H&E staining of the tumor nodules in livers. **(G, H)** The percentages of tumor infiltrated hCD45 cells **(G)**, T cells **(H** left**)** and Treg cells (**H** right**)** as determined by flow cytometry. **(I)** The percentages of M-MDSCs in bone marrow as determined by flow cytometry. As Statistical Analysis in Methods stated: * indicates p < 0.05; ** indicates p < 0.01.

We further studied LAIR1 blockade in human tumor development in humanized mice. Based on our experience in employing humanized mice to study cancer development (26–29), we established a new humanized mouse model to investigate the role of LAIR1. We first transplanted human CD34^+^ cells into NSG-SGM3 mice (Jax #013062, which are NSG mice with transgenic expression of human SCF, GM-CSF, and IL-3 to enable better engraftment of human myeloid cells); after 3 months we detected significant human CD45^+^ chimerism (with human myeloid and T cells) in the blood of mice (**Supplementary Figure 12A**). We then *subcutaneously* implanted MDA-MB-231 human breast cancer cells. After tumor size reached 50-60 mm^3^, we started to treat the mice with control or h219-LLG antibody. h219-LLG significantly impaired the cancer growth (**Figure 7A**). While having no significant effect on primary tumor growth, h219-LLG clearly decreased the liver metastasis that was observed at 9 weeks post-tumor implantation (**Figures 7B-F**). Consistently, h219 elevated the percentages of total human CD3^+^ T cells and decreased CD4^+^CD25^+^CD127^low/-^ Tregs and phenotypic M-MDSC populations in tumors (**Figures 7G-I**, **Supplementary Figures 12B-E**). Because the only cells that expressed LAIR1 in these mice were human immune cells (**Supplementary Figure 12F** **)**, our result suggests that the anti-tumor effect of the anti-LAIR1 was from inhibiting LAIR1 signaling in the human immune cells. Together, we demonstrate that the anti-LAIR1 antagonist antibody inhibited tumor development in mice.

## Discussion

ITIM-containing immune inhibitory receptors including LAIR1 are becoming attractive immune checkpoint targets in cancer and immune-related diseases (2). The roles of LAIR1, a classical ITIM-containing receptor, have been studied in hematologic malignancies and solid cancer (5, 9, 13, 14, 22–25). Here we report our development of an anti-LAIR1 antagonist antibody that shows anti-tumor immune activation activity. Using several functional assays including multiple ligand-inducible LAIR1 chimeric receptor reporter assays, the analyses of immune cell activities of human cells including those from cancer patients, human LAIR1 transgenic mice, and the humanized xenograft tumor model, we demonstrate that active blocking LAIR1 signaling with the LAIR1 antagonist antibody can activate anti-tumor function of multiple immune cells and inhibit tumor development.

To date, no antagonist activity of an anti-LAIR1 antibody has been published. The cross-linking of LAIR1 in NK cells by DX26, an anti-LAIR1 antibody, delivered a negative signal to NK cells that is capable of decreasing target cell lysis by both resting and activated NK cells *in vitro* (3). The cytolytic activity of freshly isolated T cells, was inhibited by anti-LAIR-1 mAb DX26 (47). However, it was unclear whether we could directly act on LAIR1 to block its signaling and stimulate anti-cancer immune responses. Our experience suggests it is relatively more difficult to identify antagonist anti-LAIR1 antibodies than antagonist anti-LILRB antibodies (26–29). A reason is that, unlike other similar Ig- and ITIM-containing inhibitory receptors such as LILRBs, there is only one extracellular Ig domain of LAIR1. For such a single Ig-domain containing receptor, it is easier to develop an antibody to be able to physically crosslink the Ig domain leading to activation of the receptor than to develop an antibody that induces a certain conformation of the receptor so as to deactivate its signalling.

In this study, we developed a number of criteria to screen the antagonist antibodies. 1) The antibody should have a high binding affinity to human LAIR1, and does not bind to other receptors including GPVI, LILRB1-5, LILRA1-6, or mouse LAIR1. 2) The antibody should block LAIR1 activation induced by various ligands, including in conditions that may enable crosslinking of the antibody. We tested the latter by measuring the LAIR1 blocking ability of the antibodies in the presence of K562 cells which express FcR that may crosslink Fc-containing anti-LAIR1. 3) The antibody should induce activation of relevant types of immune cells and inhibit the activity of immunosuppressive myeloid cells from cancer patients *in vitro*. 4) The antibody should inhibit tumor growth in human receptor-transgenic mice and in xenografted humanized mice. Such principles may apply to development of antagonist antibodies against other inhibitory receptors for therapeutic purposes.

LAIR2, a soluble ortholog of LAIR1 found in human but not in mouse, contains only the extracellular domain without the intracellular signaling domain. LAIR2 was reported to inhibit tumor development in mouse models (23–25). Compared to LAIR2, the anti-LAIR1 antagonist antibody acts in a different mechanism. While LAIR2 binds to collagen, it is unclear whether it binds all other LAIR1 ligands. Therefore, LAIR2 antagonizes LAIR1 activity by competitive binding to certain ligands, whereas the anti-LAIR1 antagonist antibody actively binds LAIR1 to block its signaling and thus may provide a more effective means to inhibit LAIR1-mediated immunosuppression. In addition, because LAIR2 binds to collagens that are abundant in the body, more LAIR2 than anti-LAIR1 may be needed to saturate targets in the patient. Because LAIR2 can bind to both mouse and human collagens but anti-LAIR1 only recognizes human but not mouse LAIR1, it may be easier for the recombinant LAIR2 to achieve the anti-tumor effect in humanized mice (in which both human and mouse collagens and LAIR1 exist) than anti-the LAIR1 blocking antibody. Therefore the anti-tumor effect of the anti-LAIR1 antagonist antibody in the humanized mouse model may be underestimated.

We demonstrated that anti-LAIR1 antagonist antibodies have the potential to be applicable for anti-cancer therapeutic purposes. LAIR1 is expressed on most human hematopoietic lineages. To avoid severe toxicity, it is desirable to design antibodies lacking the Fc-mediated effector functions such as ADCC and ADCP. In our experiments, the LALA-PG mutant (37) of h219 that has no Fc-mediated effector functions showed clear efficacy in promoting anti-tumor immunity. We propose to block LAIR1 signaling as an anti-cancer immunotherapy.

## Data availability statement

The data presented in the study are deposited in the GEO repository, accession number 212516.

## Ethics statement

The animal study was reviewed and approved by IACUC of UT Southwestern.

## Author contributions

JJX, XG, MD, and HC performed experiments and analyzed data. The order of co-first authors was determined by the number and scope of experiments. XL, RH, YH, and BZ provided technical support. YC, ZK, and LT supported antibody screening and production. CL provided patient samples and data. KC, LX, and JX performed scRNAseq analysis. TH provided reagents. XCL provided advice, and NZ, ZA and CCZ supervised the study. JJX, XG, and CCZ drafted the manuscript. All authors contributed to the article and approved the submitted version.

## Funding

This work was supported by National Cancer Institute (1R01 CA248736, 1R01 CA263079-01A1, 5P30CA142543, R01DK111430, R01CA230631, and R01CA259581), Department of Defense (ME190050), the Cancer Prevention and Research Institute of Texas (RP220032, RP150551, and RP190561), the Welch Foundation (AU-0042-20030616), Leukemia & Lymphoma Society (6629-21), and Immune-Onc Therapeutics, Inc. (Sponsored Research Grant #111077). JX is a Scholar of The Leukemia & Lymphoma Society (LLS).

## Acknowledgments

We thank the technical support from Wei Xiong and Hui Deng, and advice and comments from Caroline Bonnans, Maria Costa, and Krista McCutcheon for the study.

## Conflict of interest

JJX, XG, XCL, NZ, ZA, CCZ, and XCL were inventors of a patent application covering anti-LAIR1 antibodies and their uses. NZ, ZA, and CCZ hold equity in and have Sponsored Research Agreements with Immune-Onc Therapeutics, Inc. XCL and TH are employees and hold equities of Immune-Onc Therapeutics, Inc.

The remaining authors declare that the research was conducted in the absence of any commercial or financial relationships that could be construed as a potential conflict of interest.

## Publisher’s note

All claims expressed in this article are solely those of the authors and do not necessarily represent those of their affiliated organizations, or those of the publisher, the editors and the reviewers. Any product that may be evaluated in this article, or claim that may be made by its manufacturer, is not guaranteed or endorsed by the publisher.

## References

[1] Wei SC Duffy CR Allison JP . Fundamental mechanisms of immune checkpoint blockade therapy. Cancer Discov (2018) 8:1069–86. doi: 10.1158/2159-8290.CD-18-0367 30115704

[2] Deng M Chen H Liu X Huang R He Y Yoo B . Leukocyte immunoglobulin-like receptor subfamily b (LILRB): therapeutic targets in cancer. Antib Ther (2021) 4:16–33. doi: 10.1093/abt/tbab002 33928233 PMC7944505

[3] Meyaard L Adema GJ Chang C Woollatt E Sutherland GR Lanier LL . LAIR-1, a novel inhibitory receptor expressed on human mononuclear leukocytes. Immunity (1997) 7:283–90. doi: 10.1016/S1074-7613(00)80530-0 9285412

[4] Meyaard L . LAIR and collagens in immune regulation. Immunol Lett (2010) 128:26–8. doi: 10.1016/j.imlet.2009.09.014 19836418

[5] Kang X Kim J Deng M John S Chen H Wu G . Inhibitory leukocyte immunoglobulin-like receptors: Immune checkpoint proteins and tumor sustaining factors. Cell Cycle (2016) 15:25–40. doi: 10.1080/15384101.2015.1121324 26636629 PMC4825776

[6] Lebbink RJ de Ruiter T Adelmeijer J Brenkman AB van Helvoort JM Koch M . Collagens are functional, high affinity ligands for the inhibitory immune receptor LAIR-1. J Exp Med (2006) 203:1419–25. doi: 10.1084/jem.20052554 PMC2118306 16754721

[7] Son M Santiago-Schwarz F Al-Abed Y Diamond B . C1q limits dendritic cell differentiation and activation by engaging LAIR-1. Proc Natl Acad Sci U.S.A. (2012) 109:E3160–7. doi: 10.1073/pnas.1212753109 PMC3503216 23093673

[8] Olde Nordkamp MJ van Eijk M Urbanus RT Bont L Haagsman HP Meyaard L . Leukocyte-associated ig-like receptor-1 is a novel inhibitory receptor for surfactant protein d. J Leukoc Biol (2014) 96:105–11. doi: 10.1189/jlb.3AB0213-092RR 24585933

[9] Keerthivasan S Senbabaoglu Y Martinez-Martin N Husain B Verschueren E Wong A . Homeostatic functions of monocytes and interstitial lung macrophages are regulated *via* collagen domain-binding receptor LAIR1. Immunity (2021) 54:1511–1526 e8. doi: 10.1016/j.immuni.2021.06.012 34260887

[10] Olde Nordkamp MJ Boross P Yildiz C Jansen JH Leusen JH Wouters D . Inhibition of the classical and lectin pathway of the complement system by recombinant LAIR-2. J Innate Immun (2014) 6:284–92. doi: 10.1159/000354976 PMC6741594 24192271

[11] Saito F Hirayasu K Satoh T Wang CW Lusingu J Arimori T . Immune evasion of plasmodium falciparum by RIFIN *via* inhibitory receptors. Nature (2017) 552:101–5. doi: 10.1038/nature24994 PMC5748893 29186116

[12] Tang X Tian L Esteso G Choi SC Barrow AD Colonna M . Leukocyte-associated ig-like receptor-1-deficient mice have an altered immune cell phenotype. J Immunol (2012) 188:548–58. doi: 10.4049/jimmunol.1102044 PMC3286132 22156345

[13] Chen Z Shojaee S Buchner M Geng H Lee JW Klemm L . Signalling thresholds and negative b-cell selection in acute lymphoblastic leukaemia. Nature (2015) 521:357–61. doi: 10.1038/nature14231 PMC4441554 25799995

[14] Kang X Lu Z Cui C Deng M Fan Y Dong B . The ITIM-containing receptor LAIR1 is essential for acute myeloid leukaemia development. Nat Cell Biol (2015) 17:665–77. doi: 10.1038/ncb3158 PMC4417000 25915125

[15] Perbellini O Falisi E Giaretta I Boscaro E Novella E Facco M . Clinical significance of LAIR1 (CD305) as assessed by flow cytometry in a prospective series of patients with chronic lymphocytic leukemia. Haematologica (2014) 99:881–7. doi: 10.3324/haematol.2013.096362 PMC4008102 24415628

[16] Poggi A Catellani S Bruzzone A Caligaris-Cappio F Gobbi M Zocchi MR . Lack of the leukocyte-associated ig-like receptor-1 expression in high-risk chronic lymphocytic leukaemia results in the absence of a negative signal regulating kinase activation and cell division. Leukemia (2008) 22:980–8. doi: 10.1038/leu.2008.21 18288129

[17] Cao Q Fu A Yang S He X Wang Y Zhang X . Leukocyte-associated immunoglobulin-like receptor-1 expressed in epithelial ovarian cancer cells and involved in cell proliferation and invasion. Biochem Biophys Res Commun (2015) 458:399–404. doi: 10.1016/j.bbrc.2015.01.127 25660999

[18] Jingushi K Uemura M Nakano K Hayashi Y Wang C Ishizuya Y . Leukocyte−associated immunoglobulin−like receptor 1 promotes tumorigenesis in RCC. Oncol Rep (2019) 41:1293–303. doi: 10.3892/or.2018.6875 30483814

[19] Wang Y Zhang X Miao F Cao Y Xue J Cao Q . Clinical significance of leukocyte-associated immunoglobulin-like receptor-1 expression in human cervical cancer. Exp Ther Med (2016) 12:3699–705. doi: 10.3892/etm.2016.3842 PMC5228450 28105100

[20] Yang LL Zhang MJ Wu L Mao L Chen L Yu GT . LAIR-1 overexpression and correlation with advanced pathological grade and immune suppressive status in oral squamous cell carcinoma. Head Neck (2019) 41:1080–6. doi: 10.1002/hed.25539 30549148

[21] Wu X Zhang L Zhou J Liu L Fu Q Fu A . Clinicopathologic significance of LAIR-1 expression in hepatocellular carcinoma. Curr Probl Cancer (2019) 43:18–26. doi: 10.1016/j.currproblcancer.2018.04.005 29776595

[22] Peng DH Rodriguez BL Diao L Chen L Wang J Byers LA . Collagen promotes anti-PD-1/PD-L1 resistance in cancer through LAIR1-dependent CD8(+) T cell exhaustion. Nat Commun (2020) 11:4520. doi: 10.1038/s41467-020-18298-8 32908154 PMC7481212

[23] Ramos MIP Tian L de Ruiter EJ Song C Paucarmayta A Singh A . Cancer immunotherapy by NC410, a LAIR-2 fc protein blocking human LAIR-collagen interaction. Elife (2021) 10:e62927. doi: 10.7554/eLife.62927 34121658 PMC8225389

[24] Vijver SV Singh A Mommers-Elshof E Meeldijk J Copeland R Boon L . Collagen fragments produced in cancer mediate T cell suppression through leukocyte-associated immunoglobulin-like receptor 1. Front Immunol (2021) 12:733561. doi: 10.3389/fimmu.2021.733561 34691040 PMC8529287

[25] Xu L Wang S Li J Li J Li B . Cancer immunotherapy based on blocking immune suppression mediated by an immune modulator LAIR-1. Oncoimmunology (2020) 9:1740477. doi: 10.1080/2162402X.2020.1740477 33457088 PMC7790510

[26] Deng M Gui X Kim J Xie L Chen W Li Z . LILRB4 signalling in leukaemia cells mediates T cell suppression and tumour infiltration. Nature (2018) 562:605–9. doi: 10.1038/s41586-018-0615-z PMC6296374 30333625

[27] Gui X Deng M Song H Chen Y Xie J Li Z . Disrupting LILRB4/APOE interaction by an efficacious humanized antibody reverses T-cell suppression and blocks AML development. Cancer Immunol Res (2019) 7:1244–57. doi: 10.1158/2326-6066.CIR-19-0036 PMC6677629 31213474

[28] Chen H Chen Y Deng M John S Gui X Kansagra A . Antagonistic anti-LILRB1 monoclonal antibody regulates antitumor functions of natural killer cells. J Immunother Cancer (2020) 8:e000515. doi: 10.1136/jitc-2019-000515 32771992 PMC7418854

[29] Wu G Xu Y Schultz RD Chen H Xie J Deng M . LILRB3 supports acute myeloid leukemia development and regulates T-cell antitumor immune responses through the TRAF2-cFLIP-NF-kappaB signaling axis. Nat Cancer (2021) 2:1170–84. doi: 10.1038/s43018-021-00262-0 PMC8809885 35122056

[30] Buchacher T Ohradanova-Repic A Stockinger H Fischer MB Weber V . M2 polarization of human macrophages favors survival of the intracellular pathogen chlamydia pneumoniae. PloS One (2015) 10:e0143593. doi: 10.1371/journal.pone.0143593 26606059 PMC4659546

[31] Zhang Y Choksi S Chen K Pobezinskaya Y Linnoila I Liu ZG . ROS play a critical role in the differentiation of alternatively activated macrophages and the occurrence of tumor-associated macrophages. Cell Res (2013) 23:898–914. doi: 10.1038/cr.2013.75 23752925 PMC3698641

[32] He Z Zhu X Shi Z Wu T Wu L . Metabolic regulation of dendritic cell differentiation. Front Immunol (2019) 10:410. doi: 10.3389/fimmu.2019.00410 30930893 PMC6424910

[33] Hiasa M Abe M Nakano A Oda A Amou H Kido S . GM-CSF and IL-4 induce dendritic cell differentiation and disrupt osteoclastogenesis through m-CSF receptor shedding by up-regulation of TNF-alpha converting enzyme (TACE). Blood (2009) 114:4517–26. doi: 10.1182/blood-2009-04-215020 19762488

[34] Zheng J Umikawa M Zhang S Huynh H Silvany R Chen BP . *Ex vivo* expanded hematopoietic stem cells overcome the MHC barrier in allogeneic transplantation. Cell Stem Cell (2011) 9:119–30. doi: 10.1016/j.stem.2011.06.003 PMC3151486 21816363

[35] Li Z Deng M Huang F Jin C Sun S Chen H . LILRB4 ITIMs mediate the T cell suppression and infiltration of acute myeloid leukemia cells. Cell Mol Immunol (2020) 17:272–82. doi: 10.1038/s41423-019-0321-2 PMC7052276 31700117

[36] Brondijk TH de Ruiter T Ballering J Wienk H Lebbink RJ van Ingen H . Crystal structure and collagen-binding site of immune inhibitory receptor LAIR-1: unexpected implications for collagen binding by platelet receptor GPVI. Blood (2010) 115:1364–73. doi: 10.1182/blood-2009-10-246322 20007810

[37] Troegeler A Lastrucci C Duval C Tanne A Cougoule C Maridonneau-Parini I . An efficient siRNA-mediated gene silencing in primary human monocytes, dendritic cells and macrophages. Immunol Cell Biol (2014) 92:699–708. doi: 10.1038/icb.2014.39 24890643

[38] Schwab R Crow MK Russo C Weksler ME . Requirements for T cell activation by OKT3 monoclonal antibody: role of modulation of T3 molecules and interleukin 1. J Immunol (1985) 135:1714–8. 3926880

[39] Ziegler SF Ramsdell F Alderson MR . The activation antigen CD69. Stem Cells (1994) 12:456–65. doi: 10.1002/stem.5530120502 7804122

[40] Tsoukas CD Landgraf B Bentin J Valentine M Lotz M Vaughan JH . Activation of resting T lymphocytes by anti-CD3 (T3) antibodies in the absence of monocytes. J Immunol (1985) 135:1719–23. 3926881

[41] Son M Porat A He M Suurmond J Santiago-Schwarz F Andersson U . C1q and HMGB1 reciprocally regulate human macrophage polarization. Blood (2016) 128:2218–28. doi: 10.1182/blood-2016-05-719757 PMC5095756 27683415

[42] Jin J Wang Y Ma Q Wang N Guo W Jin B . LAIR-1 activation inhibits inflammatory macrophage phenotype *in vitro* . Cell Immunol (2018) 331:78–84. doi: 10.1016/j.cellimm.2018.05.011 29887420

[43] Lacey DC Achuthan A Fleetwood AJ Dinh H Roiniotis J Scholz GM . And macrophage-CSF-dependent macrophage responses by *in vitro* models. J Immunol (2012) 188:5752–65. doi: 10.4049/jimmunol.1103426 22547697

[44] Abumaree MH Al Harthy S Al Subayyil AM Alshabibi MA Abomaray FM Khatlani T . Decidua basalis mesenchymal stem cells favor inflammatory M1 macrophage differentiation *in vitro* . Cells (2019) 8:173. doi: 10.3390/cells8020173 PMC6406276 30781712

[45] Solito S Marigo I Pinton L Damuzzo V Mandruzzato S Bronte V . Myeloid-derived suppressor cell heterogeneity in human cancers. Ann N Y Acad Sci (2014) 1319:47–65. doi: 10.1111/nyas.12469 24965257

[46] Bronte V Brandau S Chen SH Colombo MP Frey AB Greten TF . Recommendations for myeloid-derived suppressor cell nomenclature and characterization standards. Nat Commun (2016) 7:12150. doi: 10.1038/ncomms12150 27381735 PMC4935811

[47] Meyaard L Hurenkamp J Clevers H Lanier LL Phillips JH . Leukocyte-associated ig-like receptor-1 functions as an inhibitory receptor on cytotoxic T cells. J Immunol (1999) 162:5800–4. 10229813

