# Supplementary material for: Blocking LAIR1 signaling in immune cells inhibits tumor development
Source: Front Immunol. 2022 Sep 21;13:996026. doi: 10.3389/fimmu.2022.996026 (PMC9534319; doi:10.3389/fimmu.2022.996026)

## Supplementary Fig 1

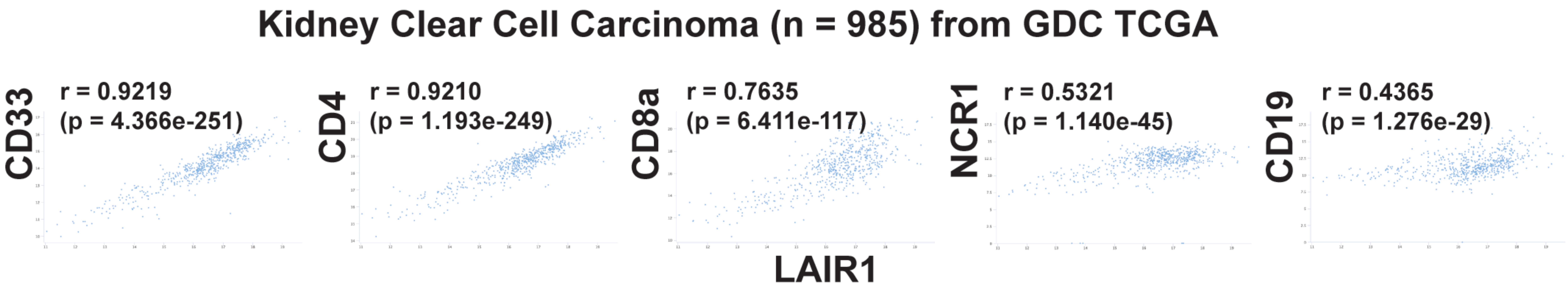

## Supplementary Fig 2

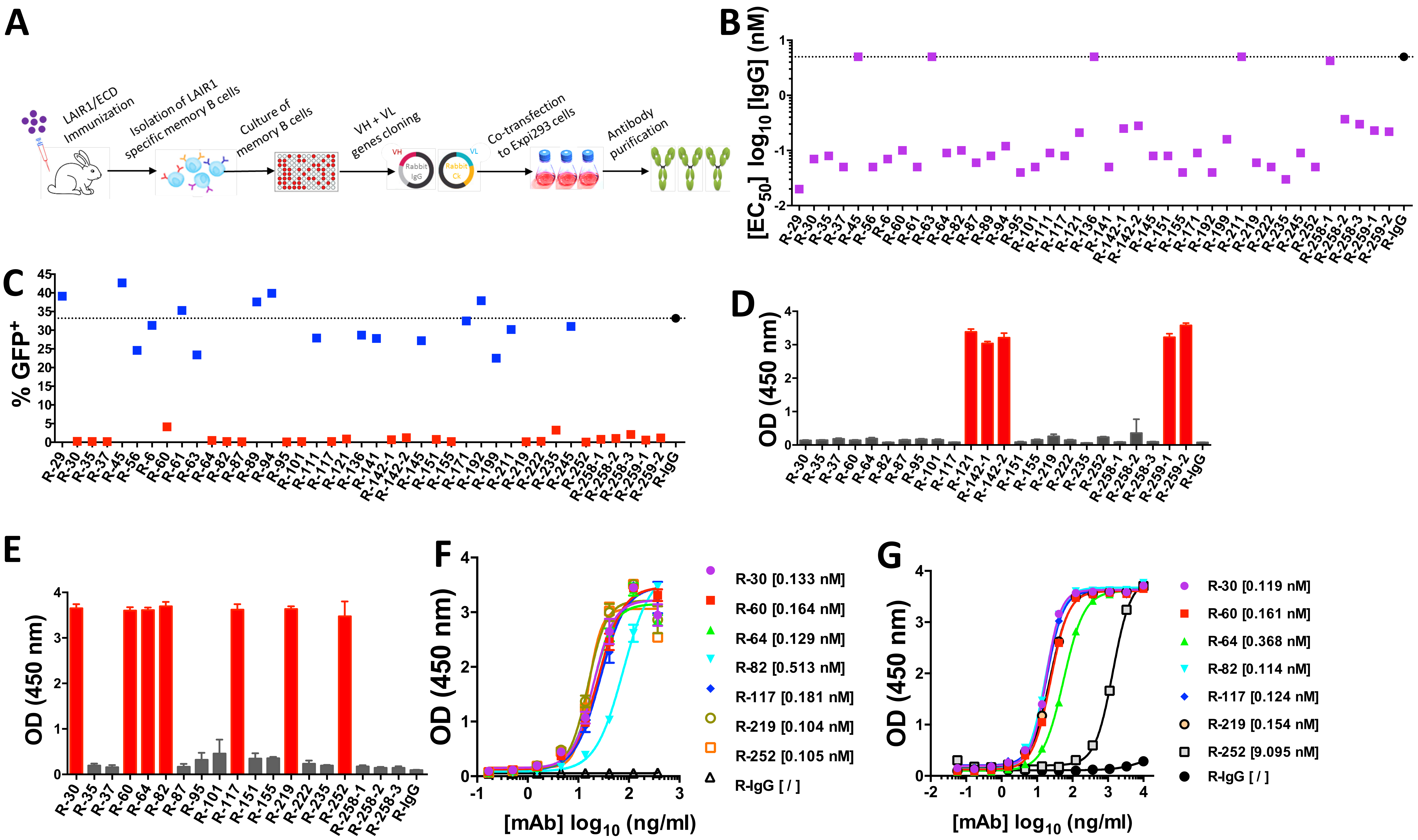

## Supplementary Fig 3

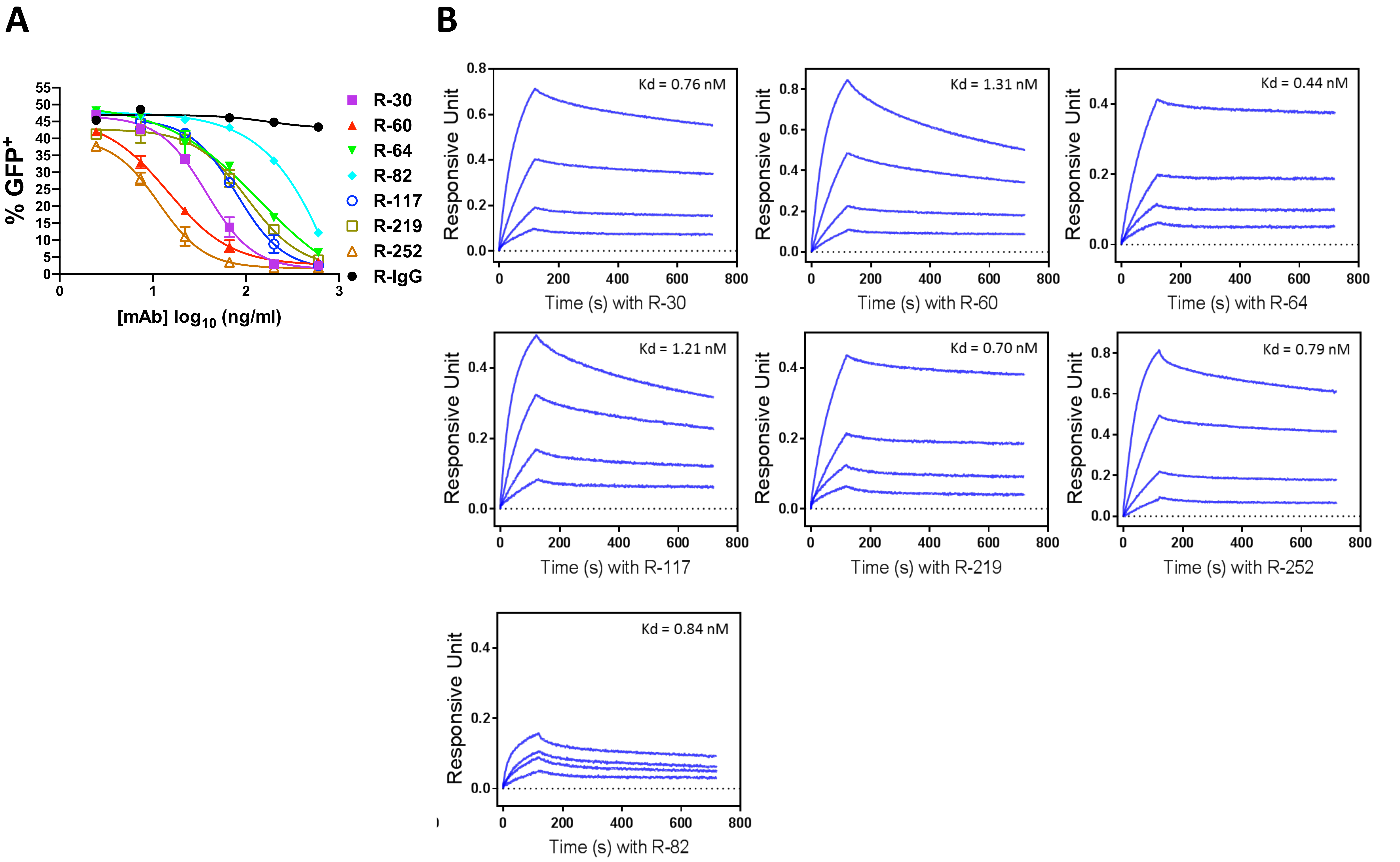

## Supplementary Fig 4

A

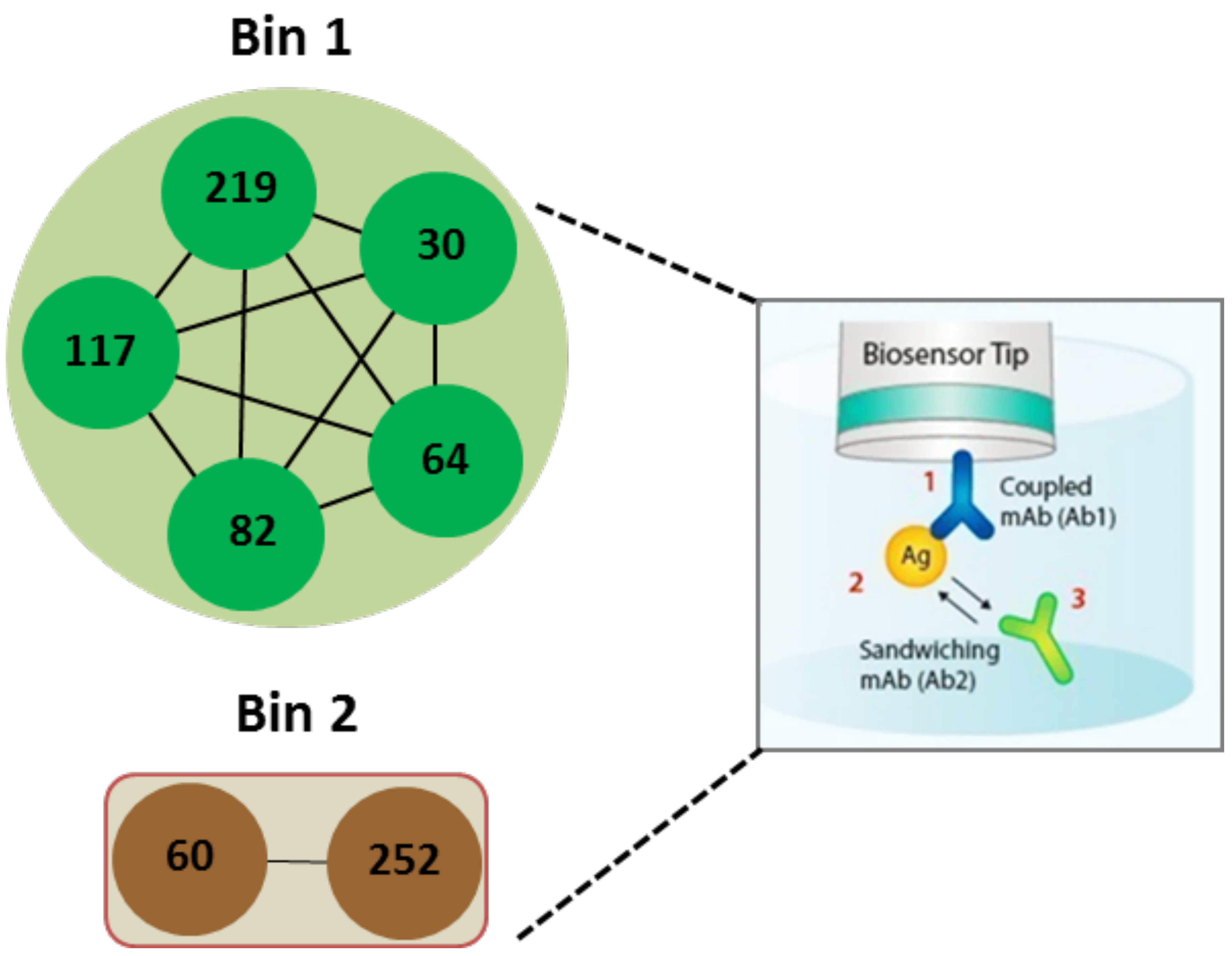


B

| | Bin A | | | | | Bin B | |
|---|---|---|---|---|---|---|---|
| | R-30 | R-64 | R-82 | R-117 | R-219 | R-60 | R-252 |
| R-30 | + | + | + | + | + | | |
| R-64 | + | + | + | + | + | | |
| R-82 | + | + | + | + | + | | |
| R-117 | + | + | + | + | + | | |
| R-219 | + | + | + | + | + | | |
| R-60 | | | | | | + | + |
| R-252 | | | | | | + | + |

# Supplementary Fig 5

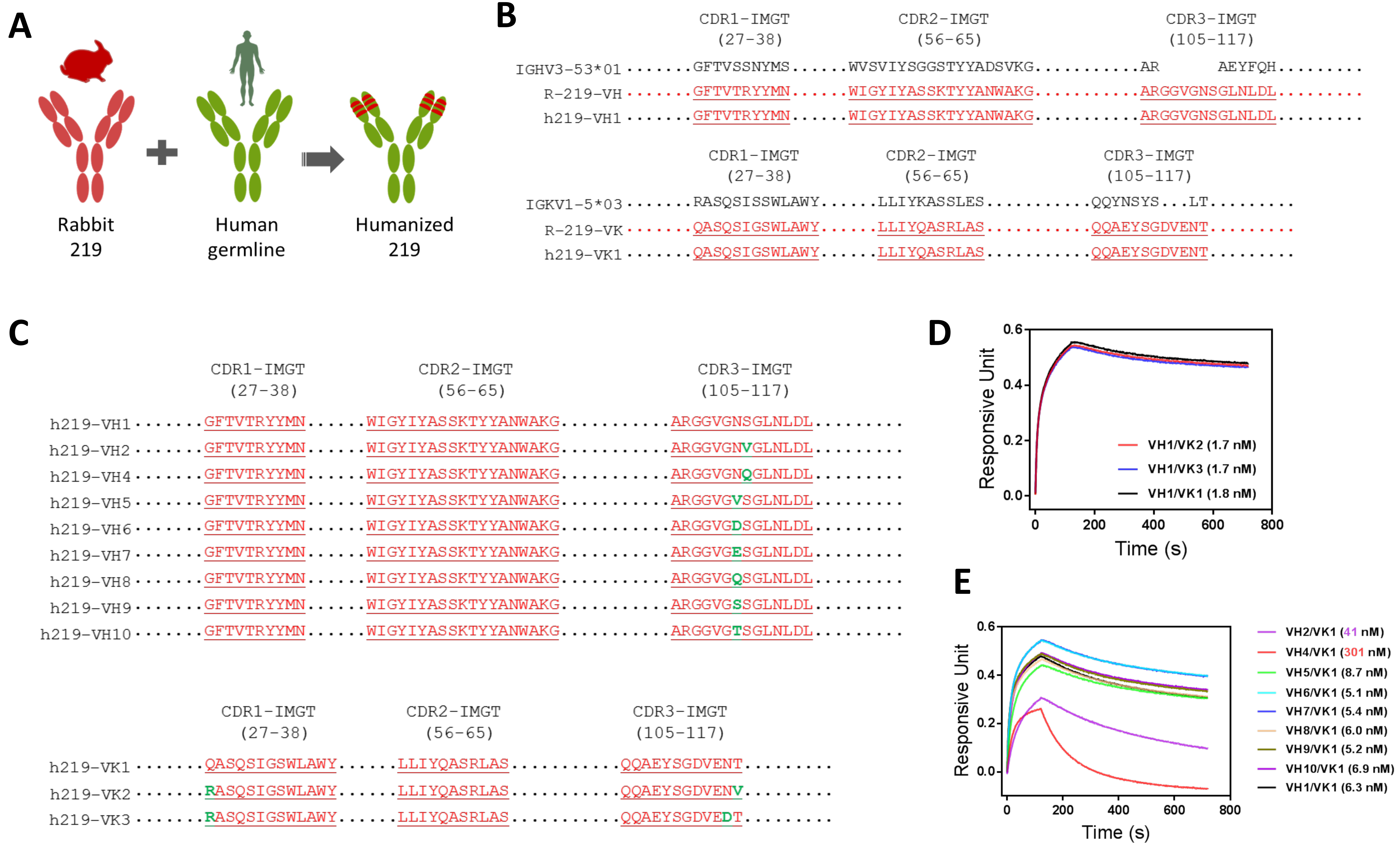

# Supplementary Fig 6

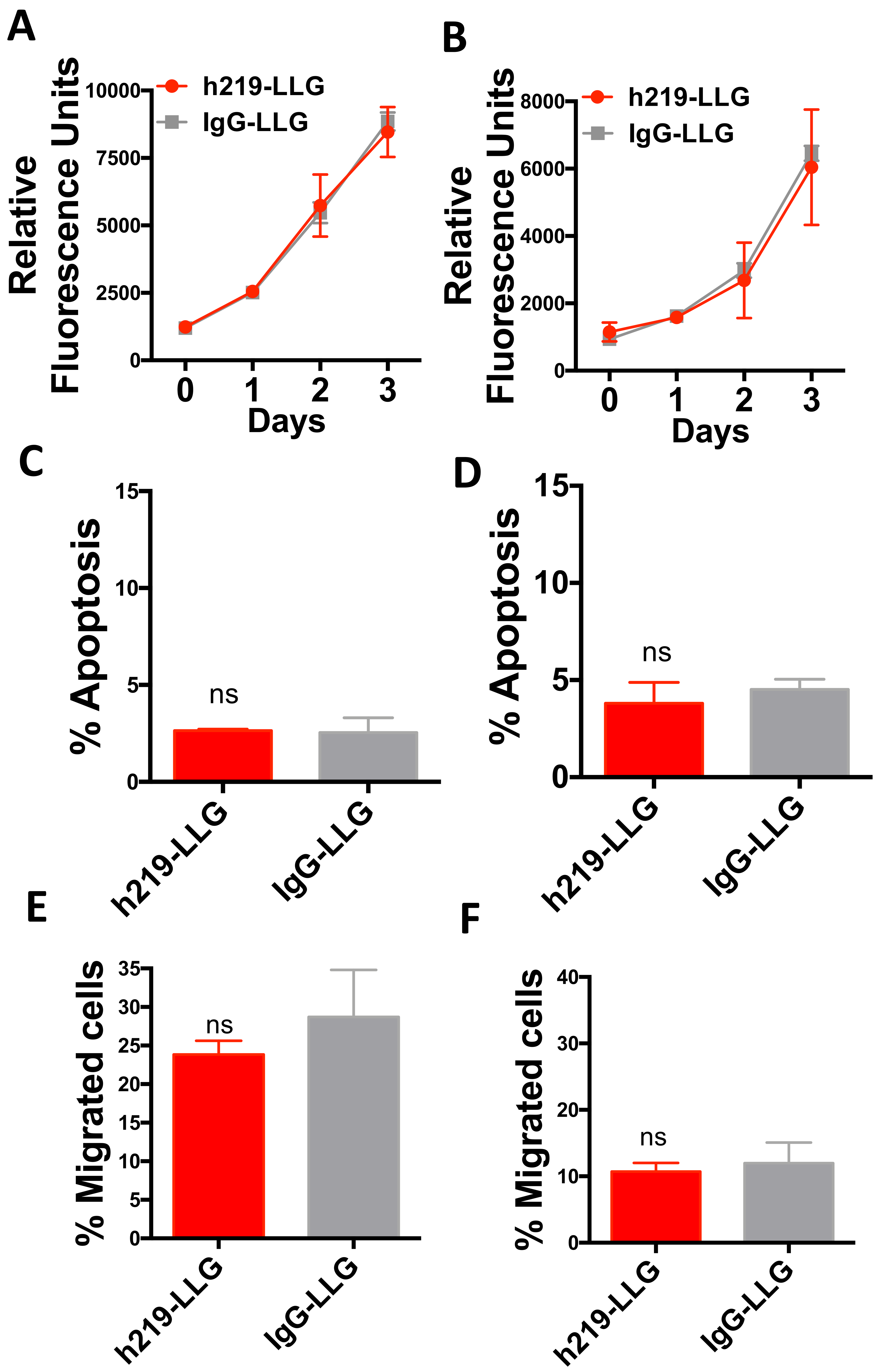

Supplementary Fig 7

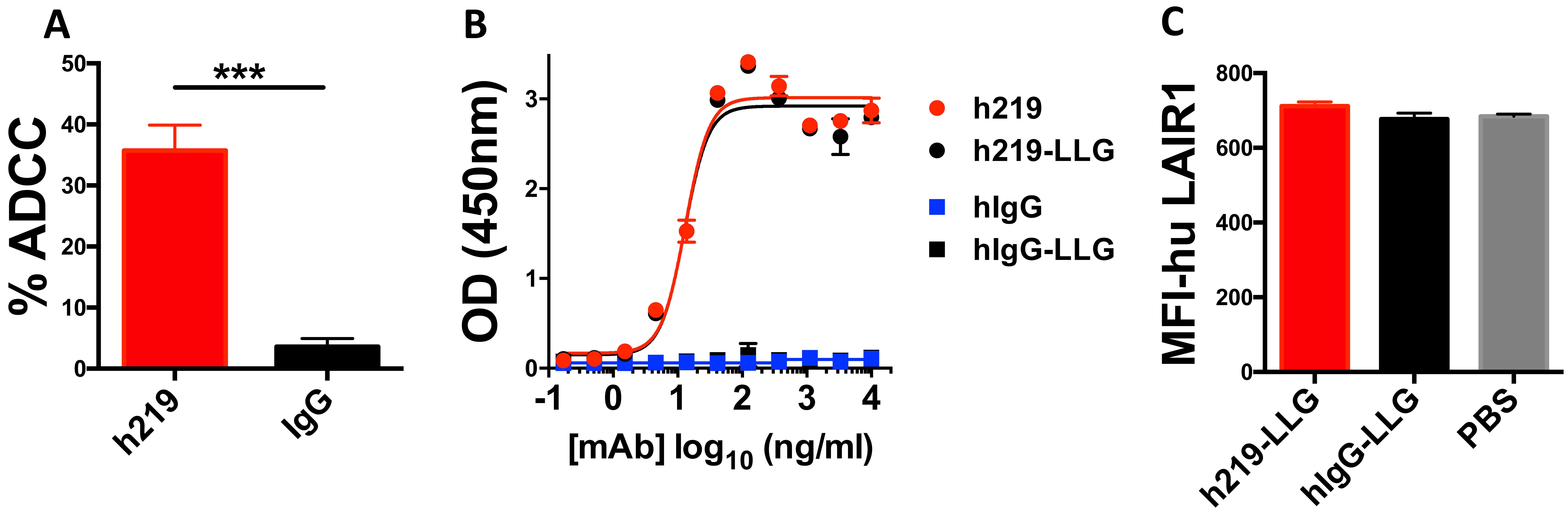

Supplementary Fig 8

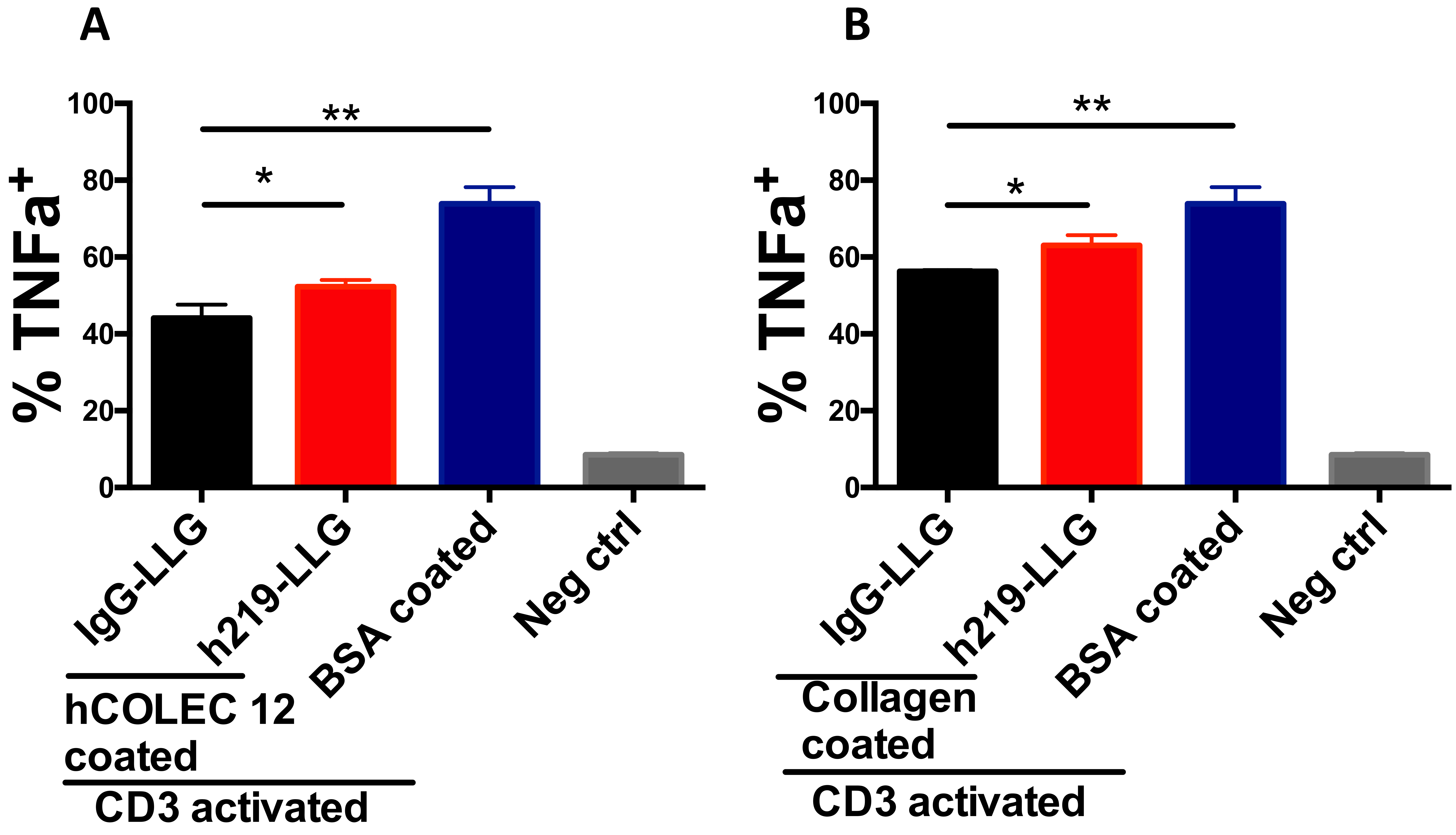

# Supplementary Fig 9

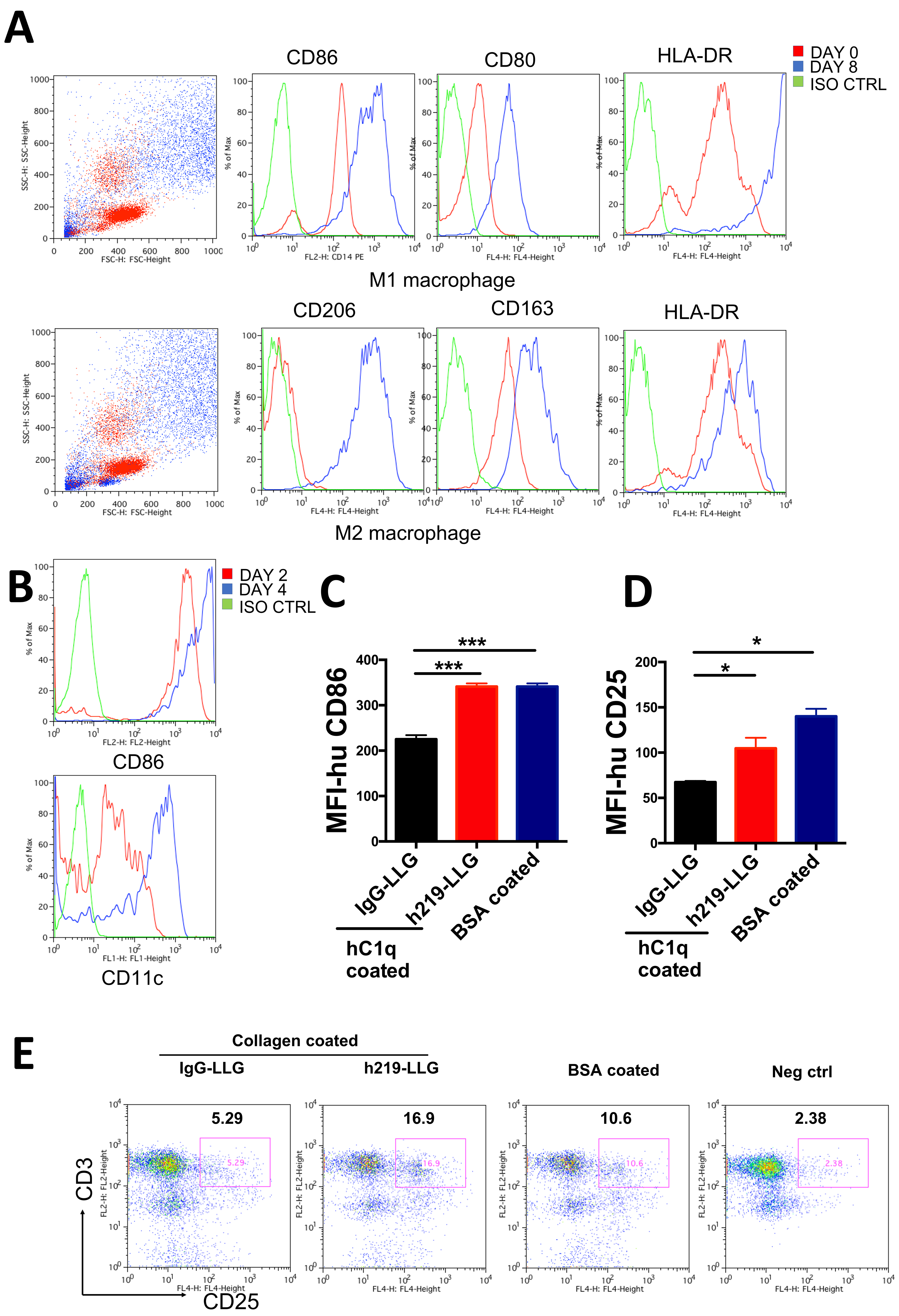

Supplementary Fig 10

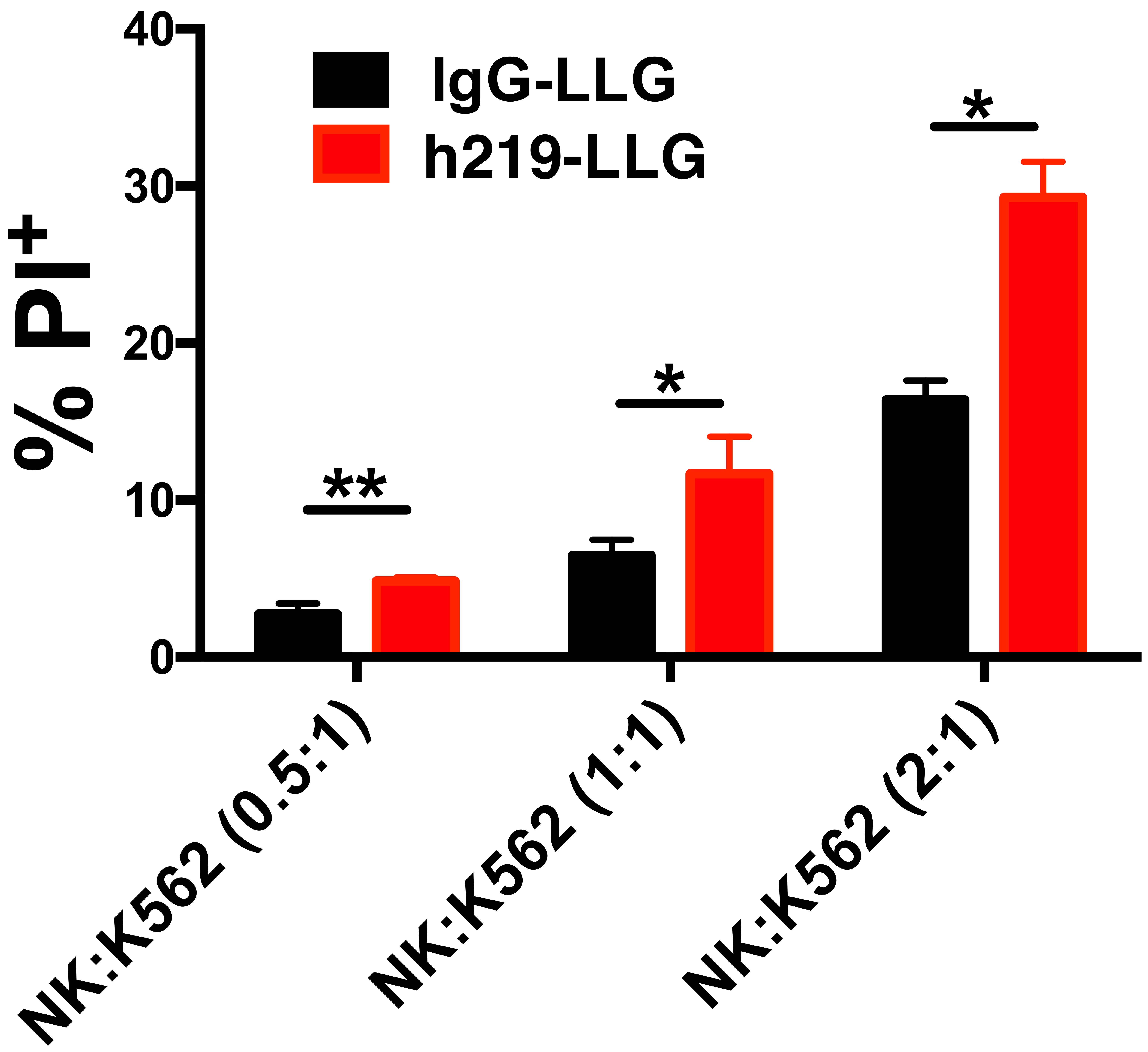

**Supplementary Fig 11**

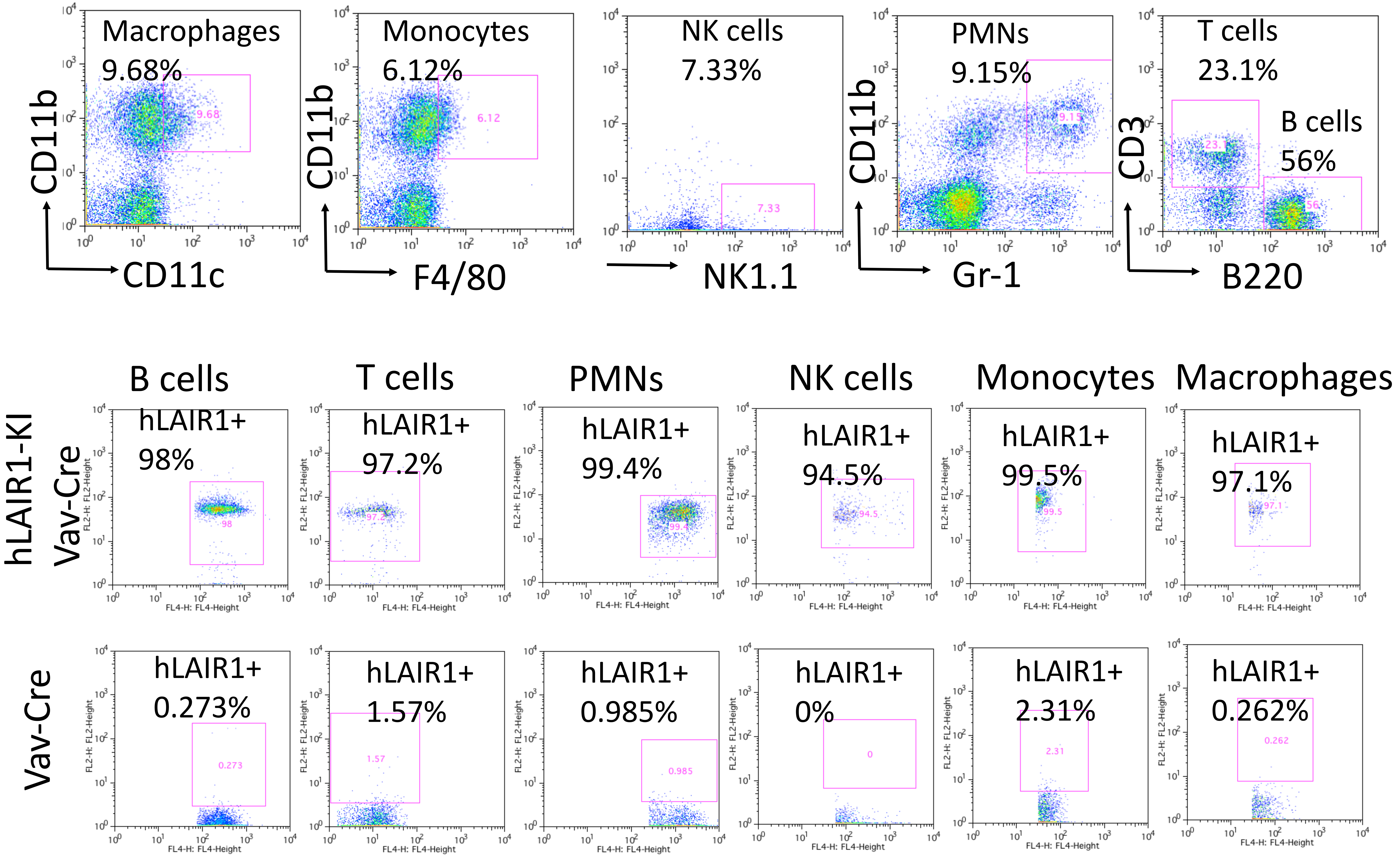

# Supplementary Fig 12

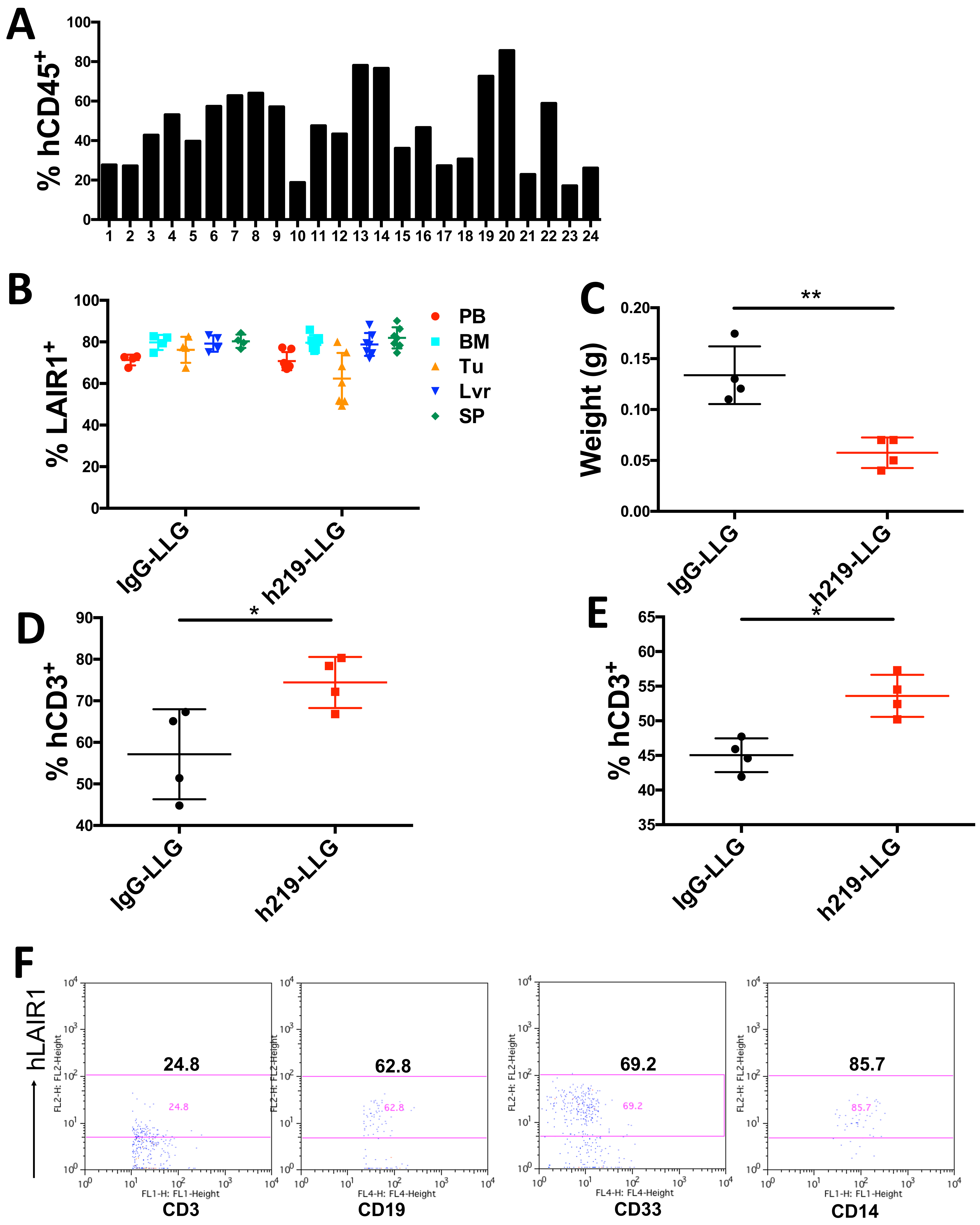

Supplement: Supplementary file 1 [file Image_1.pdf]
