# Supplementary material for: Blocking LAIR1 signaling in immune cells inhibits tumor development
Source: Front Immunol. 2022 Sep 21;13:996026. doi: 10.3389/fimmu.2022.996026 (PMC9534319; doi:10.3389/fimmu.2022.996026)
Supplement: Supplementary file 2 [file DataSheet_1.docx]

**Supplementary Materials**

**Supplementary Methods**

*Generation of LAIR1 rabbit mAbs*

Two New Zealand white rabbits were immunized subcutaneously with 0.5 mg recombinantly expressed human LAIR1 ECD protein (Sino Biological). After the initial immunization, animals were given boosters four times in a three-week interval. Serum titers were evaluated by indirect enzyme-linked immunosorbent assay (ELISA) and memory B cells were isolated after immunization was performed five times (RevMab Biosciences). A large panel of single memory B cells were collected and cultured for two weeks, and the supernatants were analyzed by ELISA (RevMab). Variable region genes from these positive single B cells were recovered by reverse transcription PCR (RT-PCR), using primers that were specific to rabbit heavy and light chain variable regions ^38^. Two rounds of PCR were performed by incorporating overlapping sequences at the 3′ and 5′ ends allowing infusion cloning of the variable regions into vectors for expression of rabbit heavy and light chains. Heavy and light chain constructs were cotransfected into human embryonic kidney freestyle 293 (HEK293F) cells using transfection reagent PEI (Sigma). After 7 days of expression, supernatants were harvested and antibodies were purified by affinity chromatography using protein A resin as we reported before (Repligen). A panel of 41 purified rabbit monoclonal antibodies was generated and used for this study.

*Identification and humanization of an anti-LAIR1 blocking mAb*

To examine whether these anti-LAIR1 rabbit mAbs block LAIR1 activation by collagen I, a functional ligand of LAIR1, we screened all 41 mAbs using a GFP-based LAIR1 chimeric receptor reporter assay ^14^. Twenty four antibodies were found to be able to neutralize the collagen I-mediated LAIR1 activation. GPVI, a protein containing 2 extracellular Ig-like domains, has different functions than LAIR1 yet possesses a similar collagen-binding property ^26^. To determine the specificity of these anti-LAIR1 antibodies, we expressed the full-length ECD of GPVI and tested its binding to the 24 LAIR1 blockers by ELISA. 19 LAIR1 blockers are specific to LAIR1 and do not cross react with GPVI. Using LAIR1 chimeric receptor reporter assay, we found that immobilized LA-219 and LA-252 had lower abilities to activate LAIR1. Because immobilized antibodies may enable crosslinking of LAIR1-extravcellular domain on the reporter cells, this is to ensure the blocking antibodies do not activate LAIR1 in any possible crosslinking event. We further measured the ligand blocking efficacy of 7 mAbs in the absence and presence of cocultured K562 cells (that express FcR that may bind to and crosslink the Fc domain of an antibody). All the 7 mAbs showed strong ligand blocking activities in the absence of K562 cells; LA-259, LA-219, and LA-252 showed potent blocking activities in the presence of K562 cells (which suggests that these antibodies can block LAIR1 activation even in the presence of a potential cellular crosslinkers, a situation that may happen in vivo when different types of FcR-expressing cells exist. To group these 7 mAbs by their binding epitopes, we performed a sandwich epitope binning assay with an Octet RED96. Two bins, Bin1 and Bin2, were identified for these 7 LAIR1 blockers. Using an Octet RED96 binding assay, we further assessed LAIR1 binding kinetics of these 7 mAbs, LA-30, LA-60. LA-64, LA-82, LA-117, LA-219 and LA-252. Kd values for binding to LAIR1 were from 0.44 nM to 1.31 nM. We selected LA-219 as the lead mAb based on its higher LAIR1 binding activity as measured by ELISA and the Octet RED96 system, its potent LAIR1/collagen blocking efficacy, and low activation potential upon crosslinking as assessed in the chimeric receptor reporter assay.

Humanization of the LAIR1 antibody was based on a CDR-grafting strategy as described previously ^38^. Briefly, CDRs in the heavy and light chains of the rabbit antibody were defined by a combination of three methods: Kabat, IMGT, and Paratome. The parental rabbit mAb and the most closely related human germline sequence were then aligned. Residues which are known not to be structurally critical and/or subjected to change during the in vivo maturation process were identified in the mutational lineage guided analysis and humanized ^42^. DNA encoding humanized VK and VH were synthesized (GenScript). The human IgG signal peptides and a Kozak sequence were engineered at the 5′ ends of the VK and VH sequences. The humanized VK and VH fragments were then cloned into human IgG1 CK and CH vectors separately. Expression, purification, and quantification of the humanized mAbs are the same as those for rabbit mAbs.

*ELISA binding assay*

Corning 96-well EIA/RIA plates were coated overnight at 4°C with human His-tagged LAIR1 or human GPVI recombinant proteins (1 μg/mL) and blocked for 2 hours at 37°C with 5% non-fat milk. After washing with PBST for 3 times, 100 μL of serial diluted LAIR1 antibodies were added and incubated for 45 minutes at 37°C. Subsequently, the plates were washed with PBST and incubated for 30 minutes with HRP-conjugated goat anti-rabbit or anti-human F(ab')_2_ (Jackson ImmunoResearch Laboratories). The immunoreactions were developed with TMB substrates (Sigma) and stopped by the addition of 2 M sulfuric acid before the plate was read at 450 nm.

*Competition ELISA assay*

Human collagen I (1 μg/ml) was coated on Corning 96-well EIA/RIA plates for overnight at 4°C, followed by blocking with 5% non-fat milk. LAIR1-hFc recombinant proteins (2 μg/ml) were incubated with LAIR1 antibodies, control IgG (5 μg/ml) or PBS at RT for 1 hour. Then the mixture were added to the pre-coated plates and incubated for 45 minutes at 37°C. Subsequently, the plates were washed with PBST and incubated for 30 minutes with HRP-conjugated goat anti-human Fc (Jackson ImmunoResearch Laboratories). The immunoreactions were developed with TMB substrates (Sigma) and stopped by the addition of 2 M sulfuric acid before the plate was read at 450 nm.

*Epitope binning with Bio-layer Interferometry (BLI)*

An Octet RED96 System, protein A biosensors, and kinetics buffer were purchased from ForteBio (Menlo Park). The epitope binning data was obtained by a BLI-based sandwich epitope binning assay performed on an 8-channel Octet RED96 instrument. First, antibodies (40 µg/mL) were loaded onto protein A biosensors for 4 minutes. The remaining Fc-binding sites on the biosensors were blocked with an irrelevant rabbit antibody (200 µg/ml) for 4 minutes, followed by soaking the biosensors in kinetics buffer for 10 seconds. The biosensors were then exposed to recombinant LAIR1 (25 µg/mL) for 4 minutes to saturate the binding site of the first antibody. Finally, the biosensors were exposed to the secondary antibodies (40 µg/mL) for 4 minutes to detect the binding. If no increased binding signal was observed with the second antibody over the binding signal of the first antibody, the pair of antibodies was classified in the same epitope bin (competitor). In contrast, if an increased binding signal was observed with the second antibody over the binding signal of the first antibody, the pair of antibodies was classified in different epitope bins (non-competitor). Bins are groups of antibodies that recognize the same binding site/area on an antigen. Protein A biosensors were reused for 10 times, and the surfaces were regenerated for 30 seconds in 100 mM glycine (pH 2.6). Raw data were processed using ForteBio’s data analysis software 7.0.

*Affinity measurement with BLI*

For antibody affinity measurement, antibody (30 µg/mL) was loaded onto the protein G biosensors for 4 minutes. Following a short baseline in kinetics buffer, the loaded biosensors were exposed to a series of recombinant LAIR1 concentrations (0.1-200 nM) and background subtraction was used to correct for sensor drifting. All experiments were performed with shaking at 1,000 rpm. Background wavelength shifts were measured from reference biosensors that were loaded only with antibody. ForteBio’s data analysis software was used to fit the data to a 1:1 binding model to extract an association rate and dissociation rate. The Kd was calculated using the ratio k_off_/k_on_.

*Annexin-V/PI apoptosis assay*

For analysis of LAIR1 antibody-induced apoptosis, 5 x 10^4^ THP-1 or MV4-11 cells were seeded in 4 replicates in 12-well plates. After 24 hours, antibodies were added to a final concentration of 20 μg/mL. Cells were treated with an irrelevant human IgG1 as isotype control. After 48 hours of incubation at 37°C in humidified air with 5% CO_2_, cells were collected, washed twice with PBS and resuspended in 150 μL of binding buffer. 5 μL of FITC-conjugated Annexin-V and PI (propidium Iodide) (BD Biosciences) was added to the cells, vortexed and incubated at RT in dark for 10 minutes. Apoptosis was measured by flow cytometry.

*ADCC assay*

THP-1 cells were incubated with anti-LAIR1 or human IgG isotype control antibodies (10 μg/ml) for 30 minutes, followed by addition of fresh human PBMC isolated NK cells as effector cells. NK cells were incubated with pre-treated THP-1 cells at an E:T ratio of 8:1 for 4 hours at 37°C. After spinning down, the supernatants were transferred to a 96-well plate to determine the amount of LDH released using LDH cytotoxicity assay kit (Pierce). Maximum release was obtained by disrupting the THP-1 cells with 0.2% Triton. Minimum release was obtained by spontaneous LDH release from the untreated THP-1 cells and NK cells.

**Supplementary Table 1. Correlation between the expression of LAIR1 and those of indicated immune cell markers in tumor tissues in several types of cancer (based on GDC TCGA database).** Pearson’s rho was shown.

|  | **Myeloid cell (CD33)** | **T cell (CD4)** | **T cell (CD8a)** | **NK cell (NCR1)** | **B cell (CD19)** |
| --- | --- | --- | --- | --- | --- |
| **Kidney clear cell carcinoma (KIRC)** | r = 0.9219 (p = 4.366e-251) | r = 0.9210 (p = 1.193e-249) | r = 0.7635 (p = 6.411e-117) | r = 0.5321 (p = 1.140e-45) | r = 0.4365 (p = 1.276e-29) |
| **Stomach cancer (STAD)** | r = 0.8390 (p = 5.724e-109) | r = 0.9313 (p = 1.633e-179) | r = 0.6764 (p = 1.014e-55) | r = 0.4789 (p = 1.035e-24) | r = 0.4241 (p = 3.386e-19) |
| **Glioblastoma (GBM)** | r = 0.9452 (p = 9.167e-85) | r = 0.9440 (p = 5.979e-84) | r = 0.3083 (p = 0.00003690) | r = 0.3675 (p = 6.577e-7) | r = 0.4571 (p = 2.622e-10) |
| **Breast cancer (BRCA)** | r = 0.8509 (p = 0.000) | r = 0.8684 (p = 0.000) | r = 0.5317 (p = 9.276e-90) | r = 0.4374 (p = 4.883e-58) | r = 0.4468 (p = 9.028e-61) |
| **Colon cancer (COAD)** | r = 0.8989 (p = 9.809e-185) | r = 0.9251 (p = 2.258e-216) | r = 0.6361 (p = 2.235e-59) | r = 0.4380 (p = 2.068e-25) | r = 0.3897 (p = 5.155e-20) |
| **Pancreatic cancer (PAAD)** | r = 0.9357 (p = 4.087e-83) | r = 0.9322 (p = 3.927e-81) | r = 0.7036 (p = 1.810e-28) | r = 0.2736 (p = 0.0001864) | r = 0.4561 (p = 9.950e-11) |
| **Lung adenocarcinoma (LUAD)** | r = 0.9114 (p = 1.034e-226) | r = 0.9083 (p = 1.244e-222) | r = 0.5053 (p = 3.205e-39) | r = 0.3765 (p = 3.882e-21) | r = 0.2278 (p = 2.516e-8) |
| **Prostate cancer (PRAD)** | r = 0.8911 (p = 2.335e-190) | r = 0.9211 (p = 8.780e-227) | r = 0.6323 (p = 8.016e-63) | r = 0.2637 (p = 3.238e-10) | r = 0.5403 (p = 4.481e-43) |
| **Ovarian cancer (OV)** | r = 0.9113 (p = 3.577e-147) | r = 0.8654 (p = 4.884e-115) | r = 0.6517 (p = 3.679e-47) | r = 0.4698 (p = 3.403e-22) | r = 0.1281 (p = 0.01253) |
| **Head & neck cancer (HNSC)** | r = 0.7972 (p = 3.206e-121) | r = 0.9037 (p = 2.526e-202) | r = 0.6677 (p = 1.056e-71) | r = 0.4589 (p = 8.724e-30) | r = 0.4189 (p = 1.301e-24) |
| **Cervical cancer (CESC)** | r = 0.8951 (p = 1.544e-109) | r = 0.9069 (p = 4.471e-117) | r = 0.6990 (p = 1.386e-46) | r = 0.4258 (p = 4.875e-15) | r = 0.4728 (p = 1.321e-18) |
| **Melanoma (SKCM)** | r = 0.7653 (p = 6.792e-92) | r = 0.8973 (p = 7.949e-169) | r = 0.8096 (p = 9.703e-111) | r = 0.5120 (p = 6.844e-33) | r = 0.5634 (p = 7.009e-41) |
| **Liver cancer (LIHC)** | r = 0.7280 (p = 3.812e-71) | r = 0.4650 (p = 4.007e-24) | r = 0.5854 (p = 2.428e-40) | r = 0.2956 (p = 5.402e-10) | r = 0.3886 (p = 9.976e-17) |

**Supplementary Table 2. Cancer patient characteristics.** The first 8 specimens were from patients with prostate cancer, which were used in MDSC/T culture. Specimen 4652 was from a patient with squamous cell carcinoma of lung.

| **Patient code** | **Treatment** | **Stage** | **Race** |
| --- | --- | --- | --- |
| 6685 | None | IIIB | White |
| 6789 | None | IIIB | White |
| 6907 | None | IVA | White |
| 7036 | Hormone therapy | IIIC | White |
| 7280 | None | IIIB | White |
| 8703 | None | IIB | White |
| 8753 | None | IIB | White |
| 8833 | None | IIIB | Black |
| 4652 | None |  | White |

**Supplementary Figure 1. A representative analysis of the correlations between the expression of LAIR1 and indicated immune cell markers in cancer tissues of kidney clear cell carcinoma patients (n = 985) in GDC TCGA database.**

**Supplementary Figure 2. The generation and screening of anti-LAIR1 rabbit mAbs.** (**A**) Single antigen-specific memory B cell isolation, culture, and cloning strategy was used to generate anti-LAIR1 rabbit mAbs. After isolation and culture of LAIR1-specific memory B cells from immunized rabbits, desired B cell supernatants were screened for binding to LAIR1 in ELISA. vH and vL genes were then cloned into rabbit IgG backbones and recombinant mAbs were produced using a transient Expi293 cell expression system. (**B**) EC_50_ of 41 anti-LAIR1 rabbit mAbs. An irrelevant rabbit antibody (R-IgG) was used as negative control. EC_50_ ≥ 5.0 nM showed as 5.0 nM. (**C**) Screening of LAIR1 antagonist mAbs by the LAIR1 chimeric receptor reporter assay. Collagen I was used as a functional ligand to activate LAIR1 reporter cells. LAIR1 antagonist mAbs are shown in red; non-blocking mAbs are shown in blue. An irrelevant rabbit antibody (R-IgG) was used as control (Black dot). (**D**) Binding of 24 LAIR1 blocking mAbs to GPVI in ELISA. (**E**) EC_50_ of 7 anti-LAIR1 mAbs to human LAIR1 in ELISA.

**Supplementary Figure 3. Antagonist activity and affinities of anti-LAIR1 mAbs.** (**A**) Human LAIR1 blocking efficacies of 7 anti-LAIR1 blocking mAbs in human LAIR1 reporter assay induced by Collagen I. **(B)** Affinities of 7 anti-LAIR1 rabbit mAbs were determined by Octet RED96. Protein A biosensors were used to capture these rabbit mAbs. The recombinant human LAIR1 protein was diluted from 45 nM to 1.6 nM. ForteBio’s data analysis software was used to fit the data to a 1:1 binding model to extract an association rate and dissociation rate. The Kd was calculated using the ratio k_dis_/k_on_.

**Supplementary Figure 4. Epitope binning of 7 anti-LAIR1 mAbs.** (**A**) Node plot of the epitope bins of 7 LAIR1 mAbs determined by Octet RED96 using a classic sandwich epitope binning assay. (**B**) Epitope binning of 7 anti-LAIR1 mAbs using a classical sandwich epitope binning assay format. “+” means antibody pair recognizing the same epitope bin.

**Supplementary Figure 5. Humanization and engineering of R-219.** (**A**-**B**) A combined KABAT/IMGT complementarity determining regions (CDR) graft strategy was used to humanize rabbit mAb R-219. (**C**) Mutations design of h219 to eliminate deamidation sites “NS” in VH and “NT” in VK. Mutated amino acid residues are shown in green. (**D**) Kds of h219 VH1 and VK combinations were determined by Octet RED96. 500 nM of human LAIR1 recombinant protein was used in this assay. (**E**) Kds of h219 VK1 and VH combinations were determined by Octet RED96. 500 nM of human LAIR1 recombinant protein was used in this assay.

**Supplementary Figure 6. h219 does not affect cell growth, apoptosis, or migration.** (**A-B**) The effect of h219-LLG or control antibodies on cell growth of THP-1 (A) and MV4-11 (B) cells as determined by AlamarBlue assay (n = 3). (**C-D**) The effect of h219-LLG or control antibodies on apoptosis of THP-1 (C) and MV4-11 (D) cells at 48 hr as determined by using FITC Annexin V Apoptosis Detection Kit (BD Biosciences) (n = 3). (**E-F**) h219-LLG did not significantly alter the transwell migration abilities of THP-1 (E) or MV4-11 (F) cells (n = 3).

**Supplementary Figure 7. LALAPG mutation does not change the binding ability of h219.** (**A**) ADCC induced by h219 or hIgG was detected by LDH cytotoxicity assay kit (Pierce). THP-1 cells and negative selected human NK cells were used a target cells and effector cells, respectively. (**B**) The bindings of h219 and h219-LLG to human LAIR1 recombinant protein in ELISA. (**C**) Surface LAIR1 on THP-1 cells were detected by flow cytometry. THP-1 cells treated with 30 μg/ml of h219-LLGor hIgG-LLG for 48 hours followed by staining with anti-LAIR1 R-94 antibody, which recognizes a different epitope than h219.

**Supplementary Figure 8**. **h219 stimulated LAIR1-regulated T cell activity**. Human PBMCs were stimulated with 1ug/ml anti-CD3 antibody (OKT3), the percentage of TNFa secreting cells under the treatment of indicated conditions were detected by intracellular staining of flow cytometry.

**Supplementary Figure 9**. **h219 stimulated LAIR1-regulated activities of monocytes and DCs**. (**A**) Flow cytometry plots showing differentiation of M1 and M2 macrophages. (**B**) Flow cytometry plots showing differentiation of dendritic cells. (**C**) CD14^+^ monocytes from fresh human PBMCs were cultured in human C1q pre-coated wells in the presence of GM-CSF and IL-4 for 2 days. CD86^+^ cells were detected. (**D**) Matured DCs under indicated conditions were mixed with allogenic T cells in the MLR assay, and the T cell activation marker CD25 were detected by flow cytometry at day 2. (**E**) Representative flow cytometry plots for the Fig. 4B experiment.

**Supplementary Figure 10.** **Inhibition of LAIR1 enhances cytotoxicity of human NK cells.** Human NK cells were pretreated with h219 and control antibodies for 48 hours before mixing with CFSE stained K562 target cells. The percentage of CFSE and PI double positive cells were detected by flow cytometry after 2 hours of co-culture.

**Supplementary Figure 11.** **Representative flow cytometry plots showing LAIR1 expression on different hematopoietic lineages in the Vav-cre LAIR1 transgenic mice.**

**Supplementary Figure 12. h219 inhibited tumor metastasis and increased T cells in humanized mice.** (**A**) The percentages of human CD45^+^ cells in blood of individual recipient mice at 12 weeks after transplantation of human cord blood CD34^+^ cells. (**B**) Human LAIR1 on hCD45^+^ cells in human CD34^+^ cell-reconstituted humanized mice was detected by flow cytometry in different organs in both h219 and IgG ctrl treated groups. (**C**) The weight of spleens. (**D-E**) The percentages of CD3^+^ cells in PB (**D**) and spleen (**E**). (**F**) Human LAIR1 surface expression on reconstituted human immune cells in liver metastatic tumor nodules.
